# Identification of succinylation-related genes in bladder cancer: integration of single-cell and transcriptomic data

**DOI:** 10.3389/fimmu.2026.1797389

**Published:** 2026-05-15

**Authors:** Jiajian Yang, Zhengyao You, Haojie Mo, Jinxian Pu, Gang Shen, Zhijun Miao

**Affiliations:** Department of Urology, The Fourth Affiliated Hospital of Soochow University, Medical Center of Soochow University, Suzhou Dushu Lake Hospital, Suzhou, Jiangsu, China

**Keywords:** bladder cancer, prognostic model, single-cell analysis, succinylation, transcriptomic, tumor microenvironment

## Abstract

**Background:**

Bladder cancer (BLCA) exhibits a poor prognosis, highlighting the urgent need for reliable prognostic genes. Although succinylation is linked to tumor progression, its role in BLCA remains understudied. This study aimed to identify and validate prognostic succinylation-related genes (SRGs) in BLCA and elucidate their impact on the tumor microenvironment (TME).

**Methods:**

SRGs were initially identified through transcriptomic sequencing of 15 paired BLCA and adjacent normal tissues (Soochow-BLCA cohort) and further refined by integrating single-cell and bulk transcriptomic data from The Cancer Genome Atlas (TCGA) and Gene Expression Omnibus (GEO) datasets. A prognostic risk model was developed using LASSO and multivariable Cox regression, incorporating clinical factors for nomogram construction. Mechanistic insights were obtained through functional enrichment, immune profiling, somatic mutation, and drug sensitivity analyses. The Scissor algorithm mapped bulk transcriptome risk signatures to single-cell resolution, enabling identification of high-risk cell subpopulations. Pseudotime and cell-cell communication analyses characterized dynamic expression patterns of the core genes. Finally, the expression profiles and functional roles of the core genes were validated using RT-qPCR and CCK-8 proliferation assays *in vitro*.

**Results:**

KCTD16, CD3D and GSDMB were identified as prognostic genes. The risk score derived from these genes, in combination with age and N stage, was incorporated into a risk model that exhibited robust predictive accuracy (AUC > 0.7). Expression of these genes differed significantly between non-muscle-invasive and muscle-invasive subtypes. The high-risk group displayed enhanced immune evasion (higher TIDE score, *p* < 0.001), while the TME appeared to be in an “inflamed yet dysfunctional” state. Single-cell analysis indicated epithelial cells as key subpopulations, with additional involvement of T cells and fibroblasts. Scissor^+^ cells were correlated with the high-risk phenotype and exhibited pseudotime-dependent expression patterns. RT-qPCR revealed significant upregulation of GSDMB and downregulation of KCTD16 and CD3D in BLCA (*p* < 0.05). Functionally, knockdown of GSDMB and KCTD16 significantly promoted T24 cell proliferation, supporting their tumor-suppressive roles.

**Conclusions:**

The succinylation-related prognostic model accurately predicts outcomes in BLCA, reveals links to immune escape and TME, and highlights the pivotal role of epithelial cells, offering potential targets for individualized therapy.

## 1 Background

Bladder cancer (BLCA) is a malignant tumor arising from the epithelial tissue of the bladder and represents one of the most prevalent malignancies of the urinary system. BLCA disease burden remains significantly higher in males than in females, which is projected to rank fourth in incidence and eighth in cancer-related mortality among estimated new cancer cases and deaths in American males for 2025 (1). Multiple risk factors contribute to BLCA pathogenesis, including environmental and genetic determinants. Tobacco smoking and occupational exposure to aromatic amines are recognized as the predominant risk factors, while alterations in genes like TP53 and iatrogenic exposures like prior cyclophosphamide treatment also play indispensable roles (2). Approximately 75% of patients are initially diagnosed with non-muscle-invasive bladder cancer (NMIBC), and the remaining 25% present with muscle-invasive bladder cancer (MIBC), which differ substantially in clinical behavior and therapeutic management (3). Transurethral resection of bladder tumor (TURBT) serves as the gold-standard method for treating NMIBC, whereas the 5-year recurrence and progression rates remain high, ranging from 12% to 85% and 2.9% to 50%, respectively (4). For patients with MIBC, the 5-year overall survival (OS) rate is below 50% even after radical surgery (3, 5), and it drops drastically to only 6% once metastasis occurs (3). These limitations highlight the urgent need to identify prognostic biomarkers associated with patient survival for guiding individualized therapy and improving clinical outcomes.

Among the various molecular mechanisms implicated in tumorigenesis, lysine succinylation has recently attracted considerable attention. Succinylation is a conserved post-translational modification widely present in both prokaryotic and eukaryotic organisms, where it regulates a range of biological processes, including oxidative metabolism, cellular signaling, and transcriptional regulation. Tumor-associated enzymes and metabolic pathways can influence chromatin and histone succinylation, thereby altering gene transcription and regulating the expression of tumor-related genes (6). Conversely, the transcriptional levels of succinylation-related genes (SRGs) determine the abundance of their encoded proteins, which in turn modulates the size of the substrate pool available for succinylation, ultimately shaping the overall succinylation activity within the cell and potentially affecting tumor progression and biological processes (7). Thereby, the biological effects of succinylated proteins are regulated by both the post-translational modification of target proteins and the transcriptional expression of SRGs, which provides a solid theoretical foundation for exploring their role in the onset, progression, and prognosis of BLCA using transcriptomic data. Existing evidence suggests that modulating the expression of key genes involved in cell cycle and apoptosis, as well as influencing chromatin and histone succinylation, may contribute to sustaining the malignant phenotype of NMIBC (8). However, the role of succinylation in BLCA remains poorly investigated. There is a scarcity of studies connecting succinylation to the clinical and biological characteristics of BLCA, and its precise biological functions in bladder tumorigenesis are largely unknown. Thus, investigating SRGs in BLCA is of great significance, as it may provide novel mechanistic insights and contribute to the development of new therapeutic and prognostic strategies.

Recent advances in single-cell RNA sequencing (scRNA-seq) have enable unprecedented resolution in dissecting tumor biology at the cellular level. Unlike transcriptomic approaches, scRNA-seq can elucidate cellular heterogeneity and reveal the functional contributions of specific cell types in disease progression (9). This high-resolution profiling is particularly valuable for uncovering the roles of SRGs across distinct cell subpopulations (10). In BLCA, however, existing studies have predominantly focused on the muscle-invasive subtype (11), leaving the landscape of succinylation at the single-cell level largely unexplored. Therefore, integrating large-scale transcriptome datasets with scRNA-seq may provide an effective strategy to reveal the prognostic and functional significance of succinylation in BLCA.

In this study, we systematically identified the succinylation-related prognostic genes in BLCA by combining bulk transcriptomic datasets with scRNA-seq analysis. We further evaluated the prognostic value of these genes, characterized their relationship with the tumor immune microenvironment, and mapped their cellular distribution at the single-cell level. Our findings provide new insights into the role of succinylation in BLCA progression and prognosis. The identified prognostic genes may serve as potential biomarkers and therapeutic targets, offering a theoretical foundation and practical guidance for personalized treatment strategies in BLCA.

## 2 Materials and methods

### 2.1 Data acquisition and preprocessing

The primary training set was obtained from The Cancer Genome Atlas (TCGA, https://portal.gdc.cancer.gov/), comprising RNA-seq data and clinical information from 416 BLCA tissues. After excluding samples with incomplete survival or clinical data, 404 BLCA samples were retained for prognostic analysis (accessed on December 1, 2024). A supplementary training set comprising transcriptomic sequencing data from 15 paired BLCA and adjacent normal tissue samples, collected from The Fourth Affiliated Hospital of Soochow University, was defined as the Soochow-BLCA cohort. The Soochow-BLCA cohort was specifically designed for differential genes screening, while the TCGA-BLCA dataset was used for the subsequent development and validation of prognostic models, and survival analysis. The validation set (GSE13507) was downloaded from the Gene Expression Omnibus (GEO, https://www.ncbi.nlm.nih.gov/geo/), containing 165 primary BLCA samples with complete survival information. scRNA-seq data (GSE135337) from 7 primary BLCA tissues and 1 adjacent normal tissue were utilized for single-cell analysis.

A list of 20 SRGs was compiled from published literatures (12, 13) (**Supplementary Table 1**).

### 2.2 Analysis of differential expression in the supplementary training set

Differential expression analysis between BLCA (n = 15) and control (n = 15) groups was performed using the R package “DESeq2” (v 1.42.0) (14), applying thresholds of an adjusted *p* < 0.05 and | log2 fold change (FC) | > 0.5. Visualization was achieved using the “ggplot2” package (v 3.4.1) (15) to generate volcano plots, in which the top 10 differentially expressed genes (DEGs), ranked by fold change, were explicitly labeled. Additionally, expression patterns of the top 1,000 DEGs (by *p*-value) were visualized via heatmaps using the R package “ComplexHeatmap” (v 2.14.0) (16).

### 2.3 Candidate genes screening

The “GSVA” package (v 1.42.0) (17) was employed to conduct single-sample Gene Set Enrichment Analysis (ssGSEA) and calculate SRG activity scores in TCGA-BLCA samples. The optimal cutoff value for stratifying high- and low-score groups was determined via the “survminer” package (v 0.4.9) (18). Kaplan-Meier (K-M) survival analysis confirmed significant prognostic differences between groups (*p* < 0.05). Subsequent differential analysis between high- and low-score groups identified SRG-associated DEGs, with a threshold of |log2 FC| > 0.5 and an adjusted *p*-value < 0.05. Venn analysis via the “ggvenn” package (v 0.1.9) (19) revealed candidate genes overlapping between DEGs and SRGs.

### 2.4 Gene function and protein network analysis

Gene Ontology (GO) and Kyoto Encyclopedia of Genes and Genomes (KEGG) analysis were carried out via the “clusterProfiler” package (v 4.2.2) (20) (*p* < 0.05). The results showed the most significant enrichment of the top 5 pathways. The STRING database (http://string-db.org) was utilized to predict interactions (confidence score ≥ 0.4, degree > 5), and the network was visualized with Cytoscape software (v 3.10.3) (21).

### 2.5 Development, assessment, and verification of the risk score model

To pinpoint genes associated with survival from the candidate genes, univariable Cox regression analysis was performed via the “survival” package (v 3.7.0) (22) on the TCGA dataset (Hazard Ratio (HR) ≠ 1). Testing of the proportional hazards (PH) assumption was performed for all models, and variables that met the PH assumption (*p* > 0.05) were retained. Genes with *p* < 0.01 were selected as significant prognostic factors.

Least absolute shrinkage and selection operator (LASSO) regression analysis was implemented via the “glmnet” package (v 4.1.8) (23) to reduce dimensionality and eliminate multicollinearity among the prognostic factors. Ten-fold cross-validation identified the optimal lambda value, and non-zero coefficient genes were retained as candidate prognostic genes. After verification of the PH assumption (*p* > 0.05), multivariable Cox regression analysis was performed using the “survival” package (v3.7.0), with subsequent stepwise regression analysis to identify independent prognostic genes. Risk scores for the prognostic genes were determined based on the constructed risk model. Here, “β” denoted the coefficient “coef”, while “x” represented the expression level of each prognostic gene.


$$\text{risk score}=\overset{\text{n}}{\underset{\text{i}=1}{\sum }}\text{βi}*\text{xi}$$

Using this formula, we calculated risk scores for all the TCGA-BLCA patients (n=404), who were then divided into high-risk group (HRG) and low-risk group (LRG) using the optimal cutpoint determined by the “survminer” package (v 0.4.9) (24). The distributions of risk scores and survival status were visualized to demonstrate the model’s discriminative capacity. Afterwards, K-M survival curves were generated using the “survminer” package (v 0.4.9), with log-rank tests assessing intergroup survival differences. Time-dependent receiver operating characteristic (ROC) curves at 1-, 2-, and 3-year were plotted using the R package “timeROC” (v 0.4) (25) to evaluate predictive accuracy. Heatmaps illustrating prognostic gene expression patterns between risk groups were created with the R package “ComplexHeatmap” (v 2.14.0) (16). To conduct external validation, the dataset of GSE13507 was analyzed. Identical risk stratification was utilized to ascertain the model’s robustness.

Furthermore, we conducted a prognostic analysis of 20 known SRGs in BLCA to explore whether the “drivers” of succinylation are themselves of prognostic significance. The PH assumption was assessed for each gene, and only those with a PH test *p* > 0.05 were included in the analysis. Genes with a *p* < 0.05 from univariable Cox regression were considered significantly associated with OS. Results were visualized as a forest plot using the R package forestplot, displaying HRs with 95% confidence intervals (CI).

### 2.6 Correlation analysis of prognostic genes and SRGs

To further investigate the potential regulatory relationships between selected independent prognostic genes and the 20 known SRGs, we performed Spearman correlation analysis on these genes using the correlate function of the R package linkET (v 0.0.7.4). The results were visualized as a correlation heatmap using the qcorrplot function. Setting |cor| > 0.3 and *p* < 0.05 as thresholds to screen for statistically significant correlations.

### 2.7 Clinical correlation analysis

To assess the diagnostic efficiency of risk scores regarding clinical indicators (e.g., age, gender, TNM stage), correlations between risk scores and the above clinical variables were analyzed. Wilcoxon tests were used to contrast the varying risk scores and prognostic genes among different clinical variables. Violin plots were generated to visualize risk score distributions across clinical variables. To explore the relationship between prognostic gene expression and tumor clinical stage, we classified T2 and T3 samples from the scRNA-seq dataset (GSE 135337) as MIBC, and Ta and T1 samples as NMIBC. A heatmap was then generated using the GroupHeatmap function in the R package SCP (v 0.5.6) to compare candidate gene expression levels between the NMIBC and MIBC groups.

### 2.8 Clinical prognostic model construction

Univariable Cox regression analysis was conducted on the risk score and several clinical variables (age, gender, TNM stage) using the “survival” (v 3.7.0) package (*p* < 0.05, HR ≠ 1, PH *p* > 0.05). The variables passed the multivariable Cox regression analysis (*p* < 0.05, PH *p* > 0.05) were regarded as independent predictive factors for prognosis. A nomogram predicting 1-, 2-, and 3-year survival probabilities was built via the “rms” package (v 6.9.0) (26). After that, the accuracy of the nomogram was evaluated with calibration curves. A closer proximity of the calibration curve to the 45-degree reference line indicates a higher predictive accuracy of the model. Time-ROC analysis was performed to evaluate the discriminative performance of the nomogram.

### 2.9 Molecular mechanism exploration

In order to investigate the biological pathways related to prognostic genes, Spearman’s rank correlation analysis was conducted between the expression profiles of prognostic genes and all other genes within the TCGA-BLCA training cohort. This analysis utilized the “psych” package (v 2.1.6) (27). Genes were prioritized according to their calculated correlation coefficients in descending order. GSEA was then carried out using the “c2.cp.kegg.v7.4.symbols.gmt” gene set collection retrieved from the Molecular Signatures Database (MSigDB, https://www.gsea-msigdb.org/gsea/msigdb) via the “clusterProfiler” package (v 4.2.2) (adjusted *p* < 0.05, |normalized enrichment score (NES)| > 1). The 5 most significantly enriched pathways associated with each gene were visualized via the “GseaVis” package (v 0.1.0) (28).

### 2.10 Tumor microenvironment and immune escape analysis

The ESTIMATE algorithm was employed for TCGA-BLCA samples to compute TME scores, including the ESTIMATE score, immune score, stromal score, and tumor purity. To evaluate immune escape potential, Tumor Immune Dysfunction and Exclusion (TIDE) scores were computed for samples in the TCGA-BLCA using the TIDE platform (https://github.com/jingxinfu/TIDEpy). The Wilcoxon test was then performed to compare TME scores and TIDE scores respectively across different risk groups.

### 2.11 Genomic mutation profiling

Somatic mutation data for TCGA-BLCA were analyzed via the “maftools” package (v 2.18.0) (29). Oncoplots were generated to display the 20 genes with the highest mutation frequencies in both HRG and LRG, where different mutation types were color-coded for distinction. Tumor mutational burden (TMB) was quantified as the aggregate count of somatic mutations detected per tumor sample. Differences in TMB between risk groups were assessed using the Wilcoxon test, and the Spearman correlation analysis was performed between TMB and the risk score.

### 2.12 Therapeutic response prediction

To forecast therapeutic response, an in silico drug sensitivity assessment was performed leveraging the Genomics of Drug Sensitivity in Cancer (GDSC) database (https://www.cancerrxgene.org/). Utilizing the “pRRophetic” package (v 0.5) (30), we projected the half-maximal inhibitory concentration (IC_50_) values of chemotherapeutic and molecularly targeted agents for TCGA-BLCA samples. The Wilcoxon test was used to compare IC_50_ values between HRG and LRG, and the top 5 drugs exhibiting the most pronounced disparities (*p* < 0.01) were selected for visualization.

### 2.13 Identification of the key cells by scRNA-seq analysis

GSE135337 was analyzed using the “Seurat” package (v 5.1.0) (31). Quality control measures were implemented to retain high-quality cells according to the following filtering parameters: (1) Cells were required to have between 200 and 6,000 detected genes (nFeature_RNA); (2) Cells with a total RNA count (nCount_RNA) exceeding 20,000 were excluded; (3) Cells with a mitochondrial gene expression percentage (percent.mt) greater than 10% were removed. Violin plots were generated to visualize the distribution of these quality control metrics (nFeature_RNA, nCount_RNA, and percent.mt) across all samples. Subsequently, the nCount_RNA and nFeature_RNA of each sample were analyzed for Pearson correlation, and the correlation scatter plots were drawn. To minimize technical variability and preserve biologically meaningful heterogeneity, 2,000 highly variable genes (HVGs) were identified via the variance stabilization transformation method implemented in the “FindVariableFeatures” function. The top 10 HVGs with the highest variability were labeled using “LabelPoints”. Data normalization and scaling were performed using the “NormalizeData” and “ScaleData”. Dimensionality reduction was achieved through principal component analysis (PCA) applied to the preselected HVGs. The optimal number of principal components (PCs) was determined by scree plot visualization, and the *p*-values of individual genes in each PC were calculated using the “JackStraw” and “ScoreJackStraw” functions (*p* < 0.05). Cell clustering analysis was performed using Uniform Manifold Approximation and Projection (UMAP) to identify distinct cell populations within the samples (resolution = 0.4). Furthermore, to obtain different types of cells, the R package “SingleR” (v 2.4.0) (32) and the CellMarker database (http://xteam.xbio.top/CellMarker/) were employed. Major cell types were identified through manual curation based on canonical marker genes. UMAP dimensionality reduction plots were generated to visualize the annotated cell types. Wilcoxon tests were conducted to assess the differential expression of prognostic genes between BLCA and control groups across various cell types (*p* < 0.05). Based on clinicopathological staging, the cellular distribution patterns of NMIBC and MIBC were delineated on the UMAP map, and the expression profiles of prognostic genes were systematically evaluated across the distinct disease subtypes.

### 2.14 Integration analysis of bulk transcriptome and single-cell data based on Scissor algorithm

To explore the association between the prognostic risk model and cell subpopulations at the single-cell level, we performed an integrative analysis of bulk transcriptome data from TCGA-BLCA and scRNA-seq dataset GSE135337 using the R package Scissor (v 2.0.0). First, the risk group information (high- and low-risk group) derived from the established prognostic model was used as the phenotype, and the FPKM matrix served as the bulk expression input. Scissor analysis was conducted using a binomial model, with the L1 regularization parameter alpha set to 0.5 and the cell selection proportion cutoff limited to 0.2. Cells from the single-cell dataset were categorized into three categories: Scissor^+^ cells positively correlated with the high-risk phenotype, Scissor^-^ cells positively correlated with the low-risk phenotype, and background cells. The transcriptional profile of Scissor^+^ cells closely resembled that of high-risk patients, suggesting their potential involvement in tumor progression, invasion, or immune evasion. In contrast, the transcriptional characteristics of Scissor^-^ cells reflected a protective state associated with favorable prognosis.

Subsequently, spatial distributions of Scissor-defined cells were visualized using UMAP plots. The absolute numbers and relative proportions of Scissor^+^ and Scissor^-^ cells within each cell type were statistically analyzed to identify associations between specific cell subpopulations and prognostic risk. All visualization analyses were implemented using the R package ggplot2.

### 2.15 Cell communication and pseudo-time analysis

Intercellular communication dynamics, analyzed to delineate cell-cell interactions, were inferred using the R package “CellChat” (v 1.6.1) (33). Ligand-receptor interactions were quantified based on scRNA-seq expression profiles (GSE135337), and interaction strength was visualized through network diagrams and heatmaps. The R package “Monocle” (v 2.30.1) (34) was employed to reconstruct developmental trajectories of key cells. Subclustering of key cells was performed using the “FindNeighbors” and “FindClusters” functions in Seurat. Pseudotime ordering was computed using the “orderCells” function, and prognostic gene expression dynamics along the trajectory were visualized.

### 2.16 Reverse transcription-quantitative PCR and cell proliferation assay

5 paired BLCA and adjacent normal tissue samples were additionally acquired from The Fourth Affiliated Hospital of Soochow University for validation of prognostic gene expression. For each sample, 50 mg of tissue was extracted and combined with 1 mL of TRIzol (Vazyme, R401-01, China). The mixture underwent thorough homogenization and grinding to ensure complete lysis. After 10 minutes of standing on ice, 200 µL of chloroform was added to extract the aqueous phase of RNA. Subsequently, an equivalent amount of chilled isopropanol was incorporated to extract the RNA. After further measurement of the RNA, the reverse transcription reaction was started immediately. The cDNA synthesis reaction was configured following the manufacturer’s protocol for HP All-in-one qRT Master Mix II RT203-Ver.1 (YoungGen, 24Y0124, China). Thereafter, 40 cycles of qPCR amplification were conducted on a CFX96 real-time fluorescence quantitative PCR device (BIO-RAD, XLFZ006, USA). The primer sequences were presented in **Supplementary Table 2**. The expression levels of prognostic genes were evaluated using the 2^-ΔΔCt^ method (35), and the resulting data were analyzed statistically and visualized with Graphpad Prism (v 10.1.2) (36).

Gene expression verification in the normal control and knockdown groups was conducted using PCR, following the same experimental procedures described above. Cell Counting Kit-8 (CCK-8) assays were subsequently performed to evaluate cell proliferation. T24 cells from the ATCC were cultured in RPMI 1640 medium (Gibco, USA) with 10% fetal bovine serum (FBS) and 1% penicillin–streptomycin at 37 °C, 5% CO2. GSDMB siRNAs and KCTD16 siRNAs were transfected into cells using Lipofectamine^®^ 3000 (Invitrogen). The siRNA duplex sequences for GSDMB and KCTD16 were presented in **Supplementary Table 3**. A cell proliferation assay was conducted using a CCK-8. Briefly, approximately 80% confluence was reached when the cells were plated in 96-well plates. In each well, different formulations were applied at varying amounts. After incubation for 24, 48, 72, and 96 hours under standard culture conditions, cells were incubated with CCK-8 reagent for 1 hour at 37 °C. The absorbance at 450 nm was measured.

### 2.17 Statistical analysis

All statistical analysis were executed utilizing R software version 4.2.2 and Graphpad Prism version 10.1.2. Correlation analysis was evaluated using Spearman’s test. The Wilcoxon test was used to evaluate paired sample comparisons, and the difference between two groups in the RT-qPCR was acquired using the t-test, with statistical significance set as *p* < 0.05.

## 3 Results

### 3.1 Differential gene expression analysis

**Figure 1:**
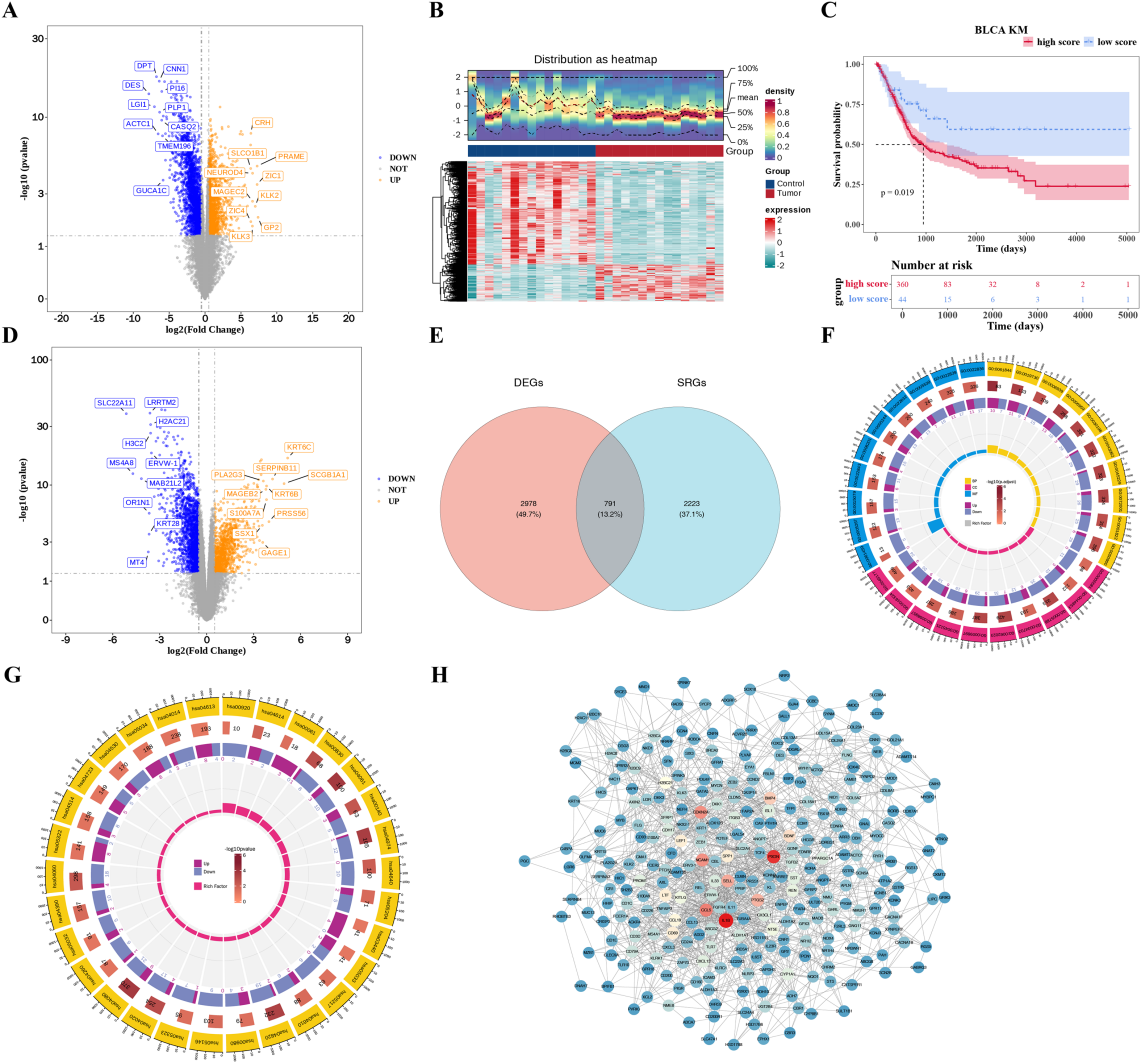
Bioinformatics analysis of SRGs in BLCA. **(A)** Volcano plot of top 10 DEGs between BLCA and adjacent normal tissues in the transcriptomic dataset of the Soochow-BLCA cohort. The x-axis represents the log2 FC, and the y-axis represents the negative log10 of the p-value. Orange dots indicate upregulated genes, and blue dots indicate downregulated genes. **(B)** Heatmap of top 1000 DEGs in the transcriptomic dataset of the Soochow-BLCA cohort. Each row represents a differentially expressed gene, and each column represents a sample from either BLCA or normal group. **(C)** K-M survival curves showing a significant disparity in OS among TCGA-BLCA patients grouped based on ssGSEA of 20 SRGs. **(D)** Volcano plot of SRG-associated DEGs in TCGA-BLCA samples. **(E)** Venn diagram showing the overlapping candidate genes between SRGs and DEGs. **(F)** Circle diagram displaying the GO enrichment analysis of candidate genes. The colors of yellow, pink and blue respectively represent BP, CC and MF. **(G)** Circle diagram showing the KEGG enrichment analysis of significant pathways. **(H)** PPI network depicting interactions among candidate genes. Each node represents a gene, and each edge represents a protein interaction between expressed genes. SRGs, succinylation-related genes; BLCA, bladder cancer; DEGs, differentially expressed genes; FC, fold change; K-M, Kaplan-Meier; OS, overall survival; ssGSEA, single-sample Gene Set Enrichment Analysis; GO, Gene Ontology; BP, biological process; CC, cellular component; MF, molecular function; KEGG, Kyoto Encyclopedia of Genes and Genomes; PPI, protein-protein interaction.

Using the transcriptomic dataset of the Soochow-BLCA cohort, 3,769 DEGs were identified between BLCA tissues (n = 15) and adjacent normal controls (n = 15) (|log_2_FC| > 0.5, *p* < 0.05), including 1,516 upregulated and 2,253 downregulated genes (**Supplementary Table 4**). Volcano plot visualization highlighted the top 10 DEGs with the largest fold changes (**Figure 1A**), while hierarchical clustering of the top 1,000 DEGs (by *p*-value) revealed distinct expression patterns between tumor and normal groups (**Figure 1B**).

### 3.2 Screening of candidate genes associated with succinylation

ssGSEA of 20 SRGs stratified TCGA-BLCA samples into high- (n = 360) and low-score (n = 44) groups (optimal cutoff = 2.098925). K-M analysis revealed statistically significant survival disparities across groups (*p* < 0.05) (**Figure 1C**). Differential expression analysis between these groups identified 3,014 SRG-associated DEGs (1,061 upregulated, 1,953 downregulated) (**Figure 1D**, **Supplementary Table 5**). Venn analysis revealed 791 candidate genes overlapping between SRGs and DEGs (**Figure 1E**), which were prioritized for further functional exploration.

### 3.3 Complex PPI network and various functional pathways of candidate genes

GO analysis of the 791 candidate genes identified 260 enriched terms (*p* < 0.05), including immune response-related biological processes (e.g., antimicrobial humoral immune response) and extracellular matrix organization. Cellular components were enriched in collagen-containing extracellular matrix and membrane microdomains, while molecular functions included potassium ion transmembrane transporter activity (**Figure 1F**). KEGG pathway analysis highlighted 18 significant pathways (*p* < 0.05), comprising viral protein-cytokine receptor interaction and calcium signaling pathway (**Figure 1G**). Moreover, a protein-protein interaction (PPI) network of 791 candidate genes (STRING confidence score ≥ 0.4) comprised 290 nodes and 1,515 edges, with candidate genes (PXDN, IL1B) exhibiting high connectivity (degree > 5) (**Figure 1H**). Overall, these findings highlighted the key biological processes involved in BLCA and provided potential targets for further research.

### 3.4 Construction and validation of prognostic risk model

**Figure 2:**
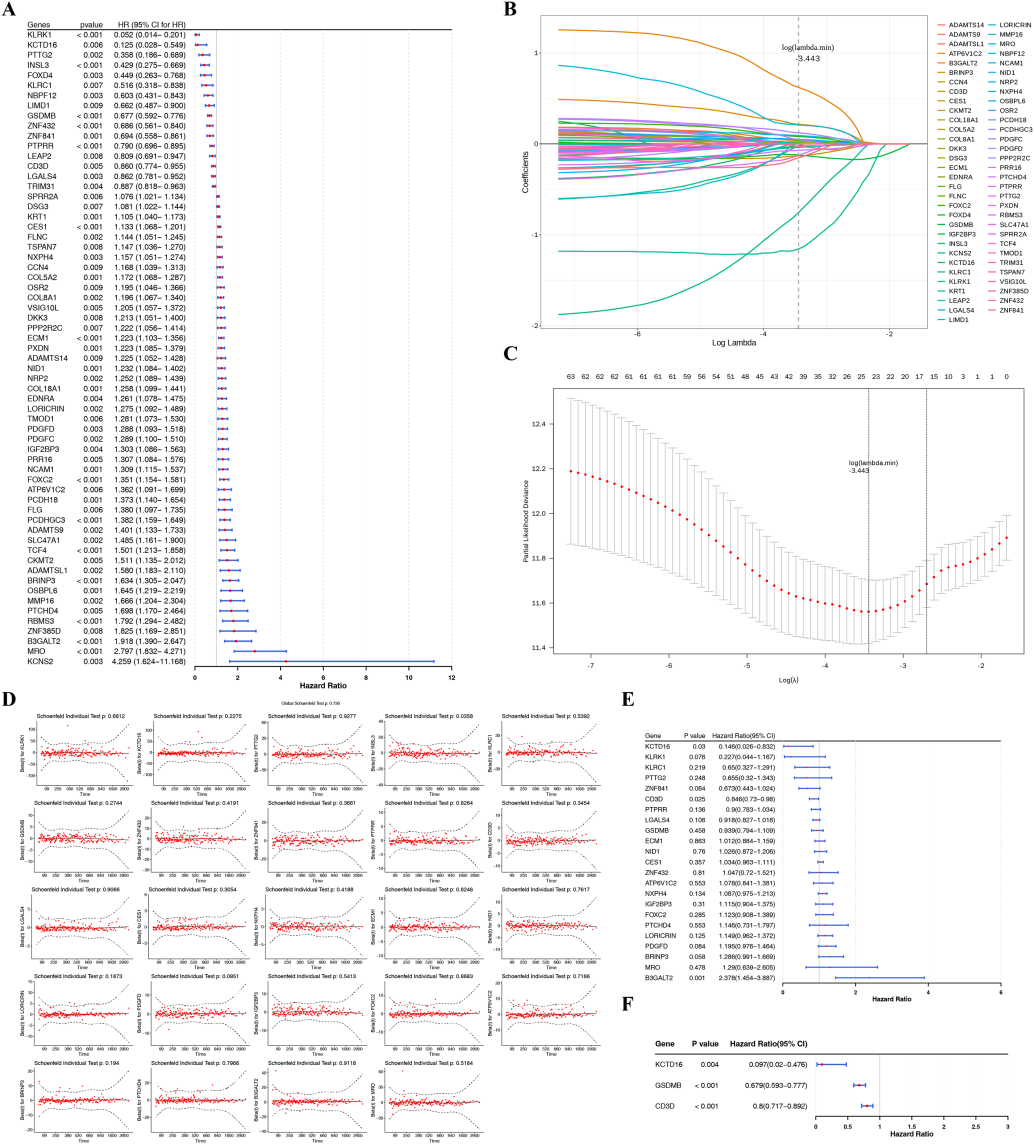
Screening of prognostic SRGs in BLCA. **(A)** Forest plot of univariable Cox regression analysis for candidate genes. **(B, C)** LASSO algorithm for prognostic genes selection, with the optimal gene number (n=24) corresponding to the lowest point on the curve. **(D)** Multivariable Cox proportional hazards assumption using Schoenfeld residuals. Black solid lines show the trendline fitted by regression coefficients over time. Red dots represent Schoenfeld residuals at each time point. Black dashed lines display the confidence intervals surrounding the trendline to illustrate estimation uncertainty. **(E, F)** Forest plot of multivariable Cox regression and stepwise selection analysis identifying independent prognostic genes. SRGs, succinylation-related genes; BLCA, bladder cancer; LASSO, least absolute shrinkage and selection operator.

Univariable Cox regression analysis and PH assumption testing (PH *p* > 0.05) for 791 candidate genes yielded a 710-variable fit model (**Supplementary Table 6**). Finally, the 63 genes significantly correlated with OS in TCGA-BLCA patients were identified (*p* < 0.01) (**Figure 2A**). LASSO regression analysis screened the above 63 genes to obtain 24 candidate prognostic genes based on the minimum lambda value (lambda.min = 0.03196892, log (lambda.min) = -3.443) (**Figures 2B, C**). Subsequent multivariable Cox regression analysis with stepwise selection was performed on the 24 genes following verification of the PH assumption, which confirmed three genes (KCTD16, GSDMB, and CD3D) as independent prognostic factors (*p* < 0.05, PH *p* > 0.05). (**Figures 2D–F**).

**Figure 3:**
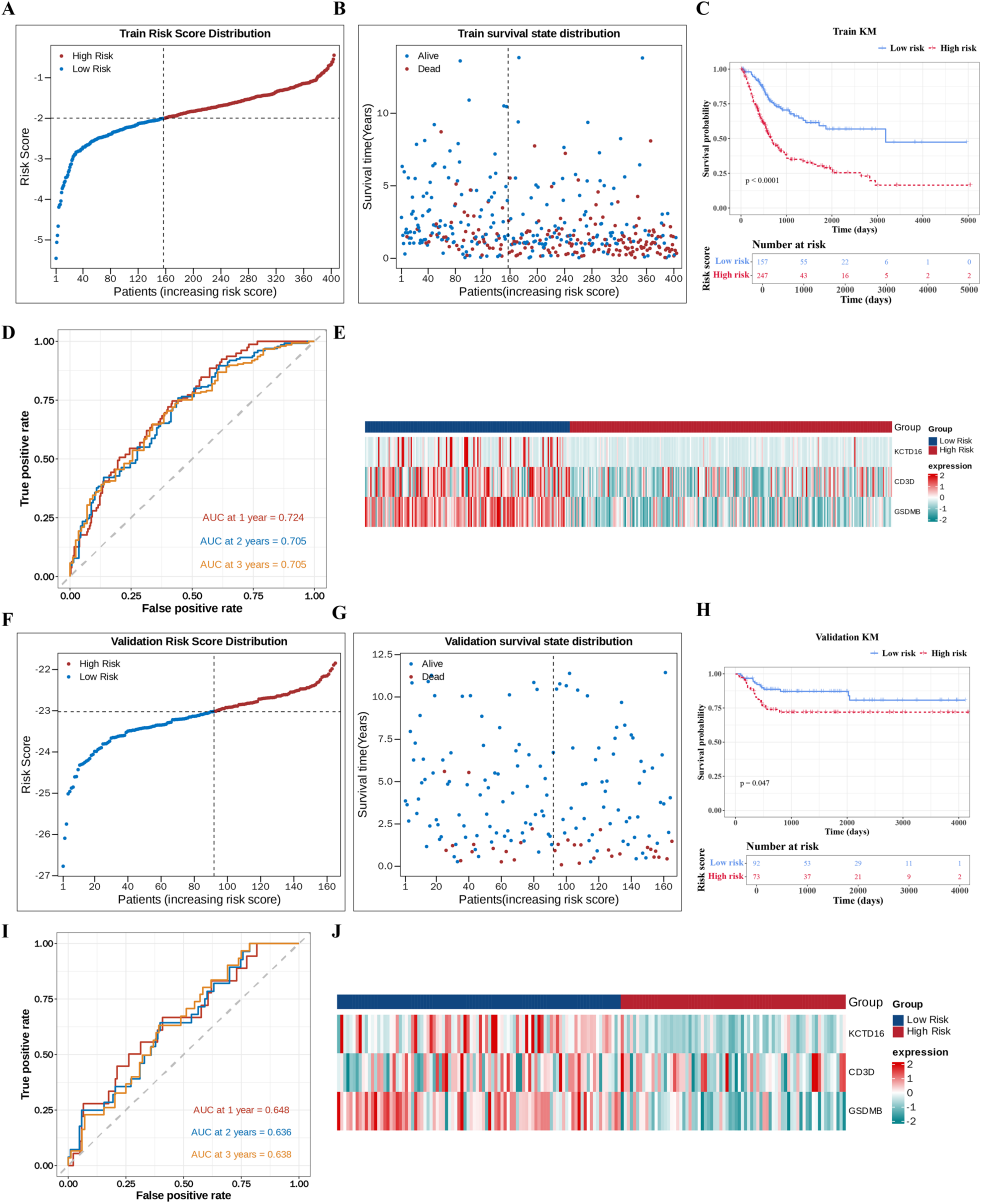
Internal and external validation of prognostic SRGs in BLCA. **(A)** Risk score distribution of TCGA-BLCA patients stratified into HRG and LRG based on the prognostic genes-derived formula. Each dot represents a sample with red for high risk and blue for low risk. **(B)** Survival status distribution of TCGA-BLCA patients stratified into HRG and LRG based on the prognostic genes-derived formula. Each dot represents a sample with red for death and blue for survival. **(C)** Kaplan-Meier survival curves showing a significant disparity in OS between risk groups. **(D)** Time-dependent ROC curves assessing robust predictive accuracy at 1–3 years. **(E)** Heatmap revealing the up-regulation and down-regulation changes of the 3 prognostic genes across risk groups. **(F–J)** External validation of the risk model in an independent validation set (GSE13507), **(F)** Risk score distribution, **(G)** Survival status distribution, **(H)** Kaplan-Meier survival curve, **(I)** Time-dependent ROC curve, **(J)** Gene expression heatmap. SRGs, succinylation-related genes; BLCA, bladder cancer; HRG, high-risk group; LRG, low-risk group; OS, overall survival; ROC, receiver operating characteristic.

A risk score formula was established based on the expression levels and regression coefficients of the prognostic genes. Subsequently, using the optimal cutpoint (-2.002354), TCGA-BLCA patients (n = 404) were stratified into HRG (n = 247) and LRG (n = 157) (**Figure 3A**). Risk score distribution plots revealed distinct separation between the groups, and survival status plots demonstrated shorter survival times in HRG (**Figure 3B**). K-M analysis confirmed significantly poorer survival outcomes in HRG compared to LRG (*p* < 0.0001) (**Figure 3C**). Time-ROC curves demonstrated robust predictive accuracy, with AUC values exceeding 0.7 for 1-, 2-, and 3-year survival (**Figure 3D**). Heatmap visualization revealed consistent downregulation of the 3 independent prognostic genes in HRG (**Figure 3E**).

To evaluate the robustness of the risk model, the same analyses were carried out in an independent validation cohort of BLCA patients (GSE13507), which were stratified into HRG (n = 73) and LRG (n = 92). The obtained results were in line with those from the TCGA-BLCA. This consistency effectively demonstrated that the prognostic model developed in this study can be effectively applied to predict clinical outcomes in BLCA (**Figures 3F–J**).

To identify independent prognostic factors among the 20 SRGs, univariable Cox regression analysis was performed for each gene, provided that the PH assumption was met (all PH tests *p* > 0.05; **Supplementary Figure 1A**). Four genes were found to be significantly associated with OS (*p* < 0.05): SIRT6 (HR = 0.578, 95% CI: 0.426–0.786), SIRT7 (HR = 0.696, 95% CI: 0.527–0.919), OXCT1 (HR = 1.163, 95% CI: 1.036–1.306), and SUCLA2 (HR = 1.369, 95% CI: 1.026–1.872) (**Supplementary Figure 1B**). Among these, high expression of OXCT1 and SUCLA2 was associated with poor prognosis (HR > 1), whereas high expression of SIRT6 and SIRT7 indicated better prognosis (HR < 1). These results suggest that upstream core regulatory genes involved in succinylation regulation possess substantial predictive potential and may play a role in regulating BLCA progression.

### 3.5 Clinical relevance of risk scores

Differences in the risk score among various clinical features were observed. Specifically, the risk score showed significant associations with clinical stage (Stage III/IV vs. I/II, *p* < 0.05), T stage (T3/T4 vs. T1/T2, *p* < 0.05), N stage (N1–3 vs. N0, *p* < 0.05), and M stage (M1 vs. M0, *p* < 0.001). No significant correlations were observed with age or gender (**Supplementary Figures 2A–F**). Given the significant correlation between risk score and tumor stage, we further analyzed the expression differences of the three core prognostic genes from NMIBC and MIBC patients in scRNA-seq dataset (GSE135337). The heatmap results showed that KCTD16 and CD3D were expressed at higher levels in NMIBC tissues, whereas GSDMB was significantly elevated in MIBC tissues (**Supplementary Figure 2G**). This expression pattern suggests that these three genes may be involved in regulating BLCA progression from non-muscle-invasive to muscle-invasive disease.

### 3.6 Establishment of the nomogram

**Figure 4:**
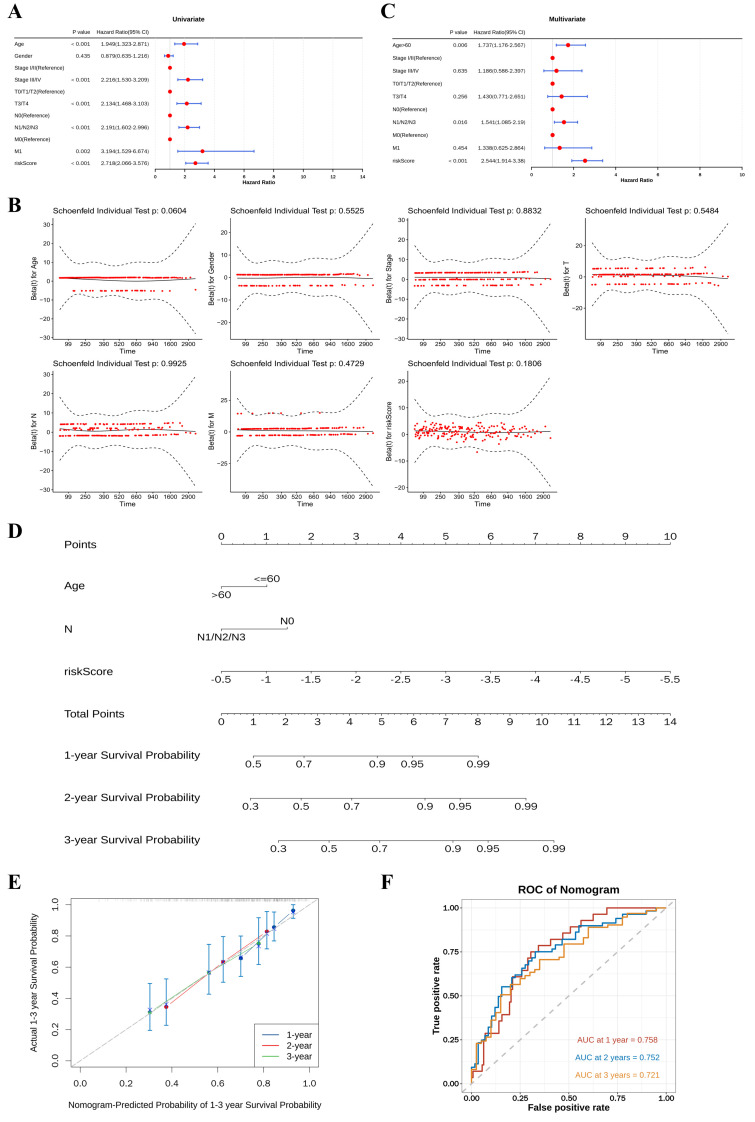
Construction and validation of the nomogram for clinical prognostic indicators related to BLCA. **(A)** Forest plot of univariable Cox regression analysis. **(B)** Univariable Cox proportional hazards assumption using Schoenfeld residuals. **(C)** Forest plot of multivariable Cox regression analysis identifying independent clinical prognostic indicators. **(D)** Nomogram for predicting the 1-, 2- and 3-year OS, incorporating the risk score and clinical variables. **(E)** Calibration plots of the nomogram for 1-, 2- and 3-year survival. **(F)** Time-dependent ROC curves for 1-, 2- and 3-year survival. BLCA, bladder cancer; OS, overall survival; ROC, receiver operating characteristic.

In the first place, a univariable Cox regression analysis was systematically performed to investigate the influence of individual factors on prognosis. Analyses indicated a significant association between factors exclude gender and adverse clinical outcomes (HR > 1, *p* < 0.05) (**Figure 4A**). Simultaneously, a PH assumption test was conducted, and the obtained p-values all exceeded 0.05, ensuring the reliability and validity of the univariable analysis results (**Figure 4B**). Multivariable Cox regression analysis was further carried out to evaluate the independent prognostic value of these factors (*p* < 0.05). Following comprehensive assessment, the 3 variables, specifically age, N stage, and risk score, were considered as independent prognostic indicators (**Figure 4C**). A nomogram incorporating risk score and clinical variables was constructed to estimate 1-, 2-, and 3-year survival probabilities, which revealed that higher total points were associated with greater probability of survival in BLCA (**Figure 4D**). Calibration curves demonstrated strong agreement between predicted and observed survival rates (**Figure 4E**). Time-dependent ROC analysis confirmed the nomogram’s discriminative capacity with AUC > 0.7 for all time points (**Figure 4F**).

### 3.7 Functional enrichment and molecular characterization of prognostic genes

**Figure 5:**
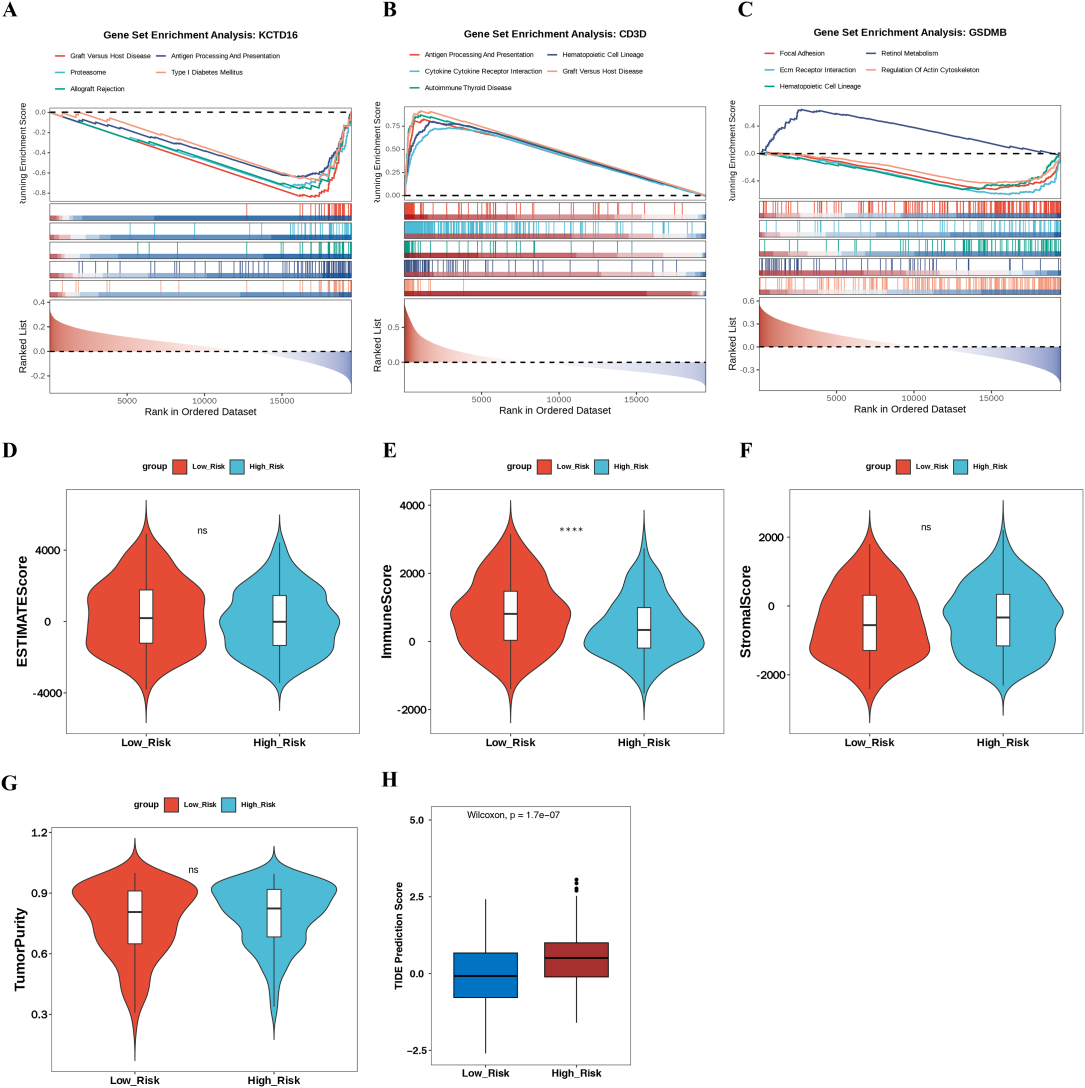
Functional enrichment and tumor microenvironment characterization of the prognostic genes. **(A–C)** GSEA showing the top 5 enriched pathways associated with prognostic genes, **(A)** KCTD16, **(B)** CD3D, **(C)** GSDMB. **(D–G)** Violin plots showing the differences in TME scores between HRG and LRG, **(D)** ESTIMATE Score, **(E)** Immune score, **(F)** Stromal score, **(G)** Tumor purity. **(H)** Box plots showing the differences in TIDE prediction scores between HRG and LRG. GSEA, Gene Set Enrichment Analysis; HRG, high-risk group; LRG, low-risk group; TME, tumor microenvironment; TIDE, Tumor Immune Dysfunction and Exclusion. ^****^p < 0.0001; ns, no significant.

GSEA analysis revealed that genes associated with KCTD16 and CD3D were co-enriched for the graft versus host disease (GVHD) pathway and the antigen processing and presentation pathway (*p* < 0.05). For GSDMB, enrichment analysis indicated its co-enrichment with CD3D in the hematopoietic cell lineage pathway (**Figures 5A–C**). These results suggested that KCTD16, CD3D, and GSDMB might have collaborated in immune responses and hematopoiesis, thereby providing a new perspective on the pathogenesis of BLCA.

### 3.8 Correlation analysis of prognostic genes and SRGs

To explore the relationship between the three core prognostic genes and succinylation regulation, we analyzed the expression correlations between these genes and 20 SRGs. GSDMB showed significant expression correlations with multiple SRGs, including the strongest positive correlation with SIRT7 (cor = 0.54, *p* < 0.001) and the strongest negative correlation with OXCT1 (cor = –0.39, *p* < 0.001) (**Supplementary Figure 3**). In contrast, the expression levels of KCTD16 and CD3D did not reach the predefined significance threshold with any of the 20 SRGs. These findings suggest that GSDMB may participate in the succinylation regulatory network of BLCA by synergizing with or antagonizing specific succinylation-related enzymes (e.g., SIRT7, OXCT1), whereas KCTD16 and CD3D may act independently of these classical regulators at the transcriptional level.

### 3.9 TME and immunotherapy response

The Wilcoxon test of TME scores between HRG and LRG revealed a significant difference in immune score across risk groups (*p* < 0.001) (**Figures 5D–G**). Although the HRG showed a significantly higher immune score, it also exhibited a markedly elevated TIDE score (p < 0.001, **Figure 5H**). This combination of high immune infiltration and high TIDE score suggests that the immune cells in HRG are predominantly dysfunctional or exhausted, leading to an “inflamed yet dysfunctional” microenvironment, which correlates with poor prognosis.

### 3.10 Genomic landscape and mutation burden

**Figure 6:**
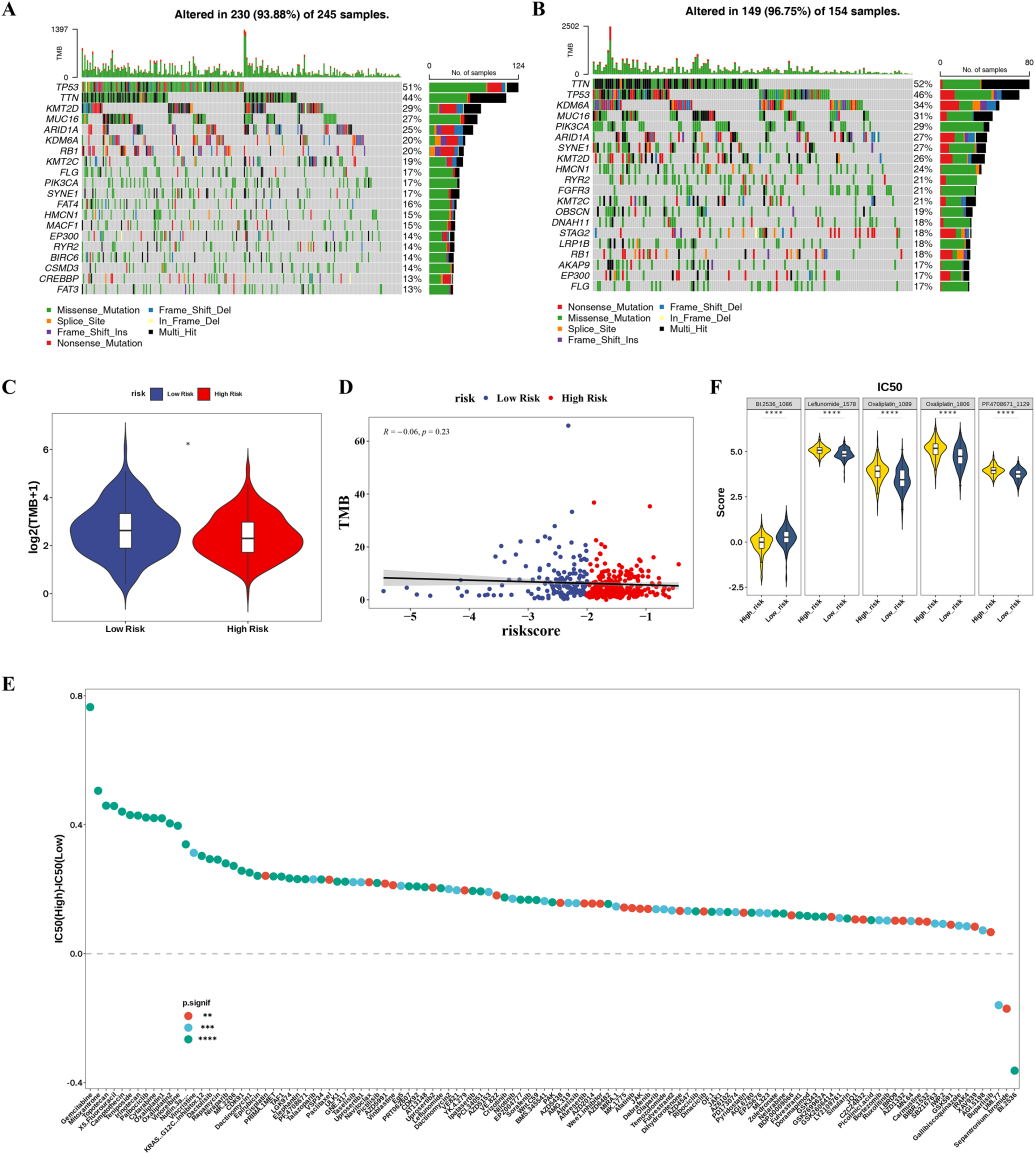
Genomic alterations and therapeutic implications of risk stratification in BLCA. **(A, B)** Mutation overview of the top 20 most frequently mutated genes in the high-risk and low-risk groups of the TCGA-BLCA dataset, **(A)** High-risk group, **(B)** Low-risk group. **(C)** Violin plots displaying the differences in TMB scores between high-risk and low-risk groups. **(D)** Scatter plot of the correlation between TMB and risk scores. **(E)** The differences in sensitivity to chemotherapeutic and targeted drugs among TCGA-BLCA patients in HRG and LRG. **(F)** Violin plots showing IC50 distributions of the top five differentially sensitive drugs. BLCA, bladder cancer; TMB, tumor mutation burden; IC50, half-maximal inhibitory concentration. *p < 0.05; **p < 0.01; ***p < 0.001; ****p < 0.0001.

Mutational profiling identified distinct patterns between risk groups: TP53 mutations predominated in HRG (51% vs. 46% in LRG), whereas KDM6A alterations were enriched in LRG (34% vs. 20% in HRG) (**Figures 6A, B**). Although TMB differed significantly between risk groups (*p* < 0.05), no direct correlation was found between TMB and the risk score (*p* = 0.23) (**Figures 6C, D**), implying that TMB may not serve as a direct surrogate marker for the specific risk score used in this study.

### 3.11 Therapeutic implications of risk stratification

Drug sensitivity analysis revealed 141 chemotherapeutic and targeted agents with differential IC_50_ values between risk groups (*p* < 0.05) (**Figure 6E**). Among the top 5 drugs showing the most pronounced IC_50_ discrepancies between groups, the distribution showed that BI-2536_1086 had a lower IC_50_ in HRG than in LRG, indicating higher sensitivity in HRG. The remaining 4 drugs had lower IC_50_ values in LRG, suggesting higher sensitivity in LRG (**Figure 6F**). This distinct pharmacological profile highlights the potential utility of risk stratification in guiding personalized treatment regimens.

### 3.12 Cellular annotation and analysis of differences between groups

**Figure 7:**
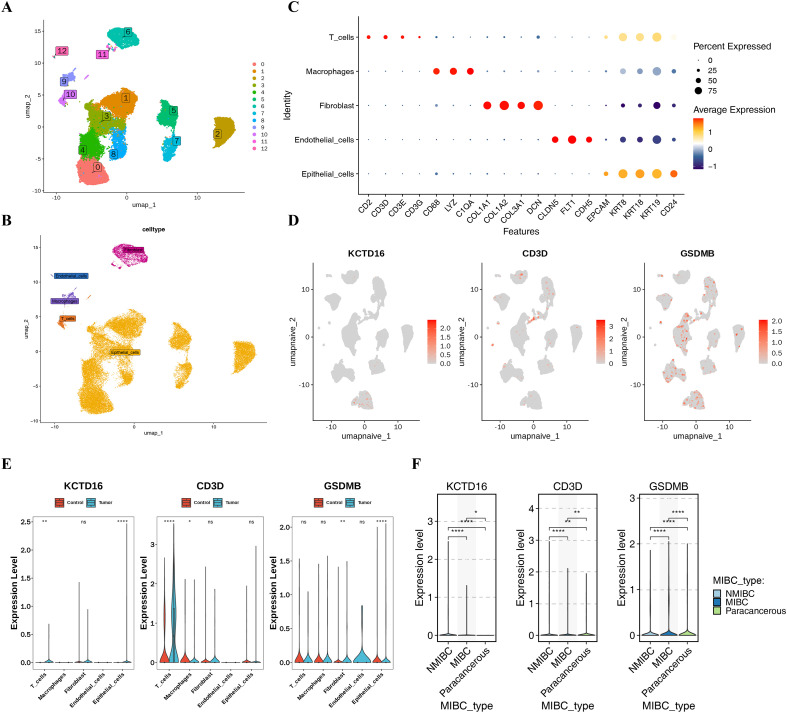
Single-cell characteristics and key cell identification in BLCA and normal tissues. **(A)** UMAP clustering diagram of 13 cell clusters. **(B)** UMAP clustering diagram of the main cell clusters annotated in BLCA and control group. **(C)** Expression point diagram of Maker genes in different cell types. **(D)** Distribution of prognostic gene expression at the single-cell level. **(E)** Wilcoxon test of prognostic genes in BLCA and control group in different cell clusters. **(F)** Expression patterns of prognostic genes in different disease types. BLCA, bladder cancer; UMAP, Uniform Manifold Approximation and Projection. ^*^p < 0.05; ^**^p < 0.01; ^****^p < 0.0001; ns, no significant.

A total of 39,899 high-quality cells (from 7 BLCA and 1 control samples in the validation cohort of GSE135337) were retained after stringent quality control (**Supplementary Figure 4A**). Strong correlation between nCount_RNA and nFeature_RNA (cor = 0.93) confirmed technical consistency across samples (**Supplementary Figure 4B**). 2,000 highly variable genes were selected for further analysis, with the top 10 genes exhibiting the most significant cell-to-cell expression changes labeled (**Supplementary Figure 4C**). Then, the top 20 PCs were chosen for subsequent analysis (**Supplementary Figures 4D, E**). They were further categorized by UMAP into 13 distinct cell clusters (resolution = 0.4) (**Figure 7A**, **Supplementary Figure 5A**). Annotation using canonical markers identified 5 major cell types: T cells, macrophages, fibroblasts, endothelial cells, and epithelial cells (**Figures 7B, C**). The UMAP map also provided an intuitive visualization of the significant and directional remodeling of the molecular expression profiles of samples as they progress from NMIBC to MIBC (**Supplementary Figure 5B**). UMAP visualization and Wilcoxon testing revealed cell type-specific expression patterns of the prognostic genes (**Figure 7D**). Both KCTD16 and GSDMB were significantly differentially expressed in epithelial cells (*p* < 0.05), which were present in sufficient numbers in BLCA and control groups and were designated as key cells (**Figure 7E**). The prognostic genes were expressed in both BLCA and adjacent normal tissues. Among them, KCTD16 and CD3D exhibited elevated expression in NMIBC, whereas GSDMB displayed the highest expression level in MIBC (**Figure 7F**).

### 3.13 The Scissor algorithm identifies cell subpopulations associated with prognostic risk

To further investigate the cellular basis of the prognostic risk model at single-cell resolution, we applied the Scissor algorithm to integrate bulk transcriptome−derived risk group information from TCGA−BLCA with the single−cell RNA−seq dataset GSE135337. Based on an L1−regularized regression model, 222 Scissor^+^ cells (positively correlated with the high−risk phenotype) and 267 Scissor^-^ cells (positively correlated with the low−risk phenotype) were identified (**Supplementary Figure 6A**). UMAP dimensionality reduction revealed partial separation between Scissor^+^ and Scissor^-^ cells in the global cell distribution space, indicating distinct transcriptomic profiles (**Supplementary Figure 6B**). Cell type composition analysis further showed that both Scissor^+^ and Scissor^-^ cells were predominantly epithelial cells, accounting for 85% and 68% of their respective totals (**Supplementary Figure 6C**). The risk phenotypes of T cells and epithelial cells exhibited marked alterations with advancing tumor stage, whereas the endothelial cell phenotype remained relatively stable, indicating that cell-type-specific risk characteristics were closely related to the invasion and progression of BLCA (**Supplementary Figure 6D**). While epithelial cells are dominant, the high-risk phenotype captured by the model is not solely determined by their intrinsic properties, but is jointly driven by transcriptional changes within immune cell populations (e.g., T cells, macrophages) and stromal components (e.g., fibroblasts). Notably, even the relatively low-abundance Scissor^+^/^-^ subsets within these non-epithelial compartments confer substantial contributions to the high-risk state.

### 3.14 Altered intercellular communication networks

**Figure 8:**
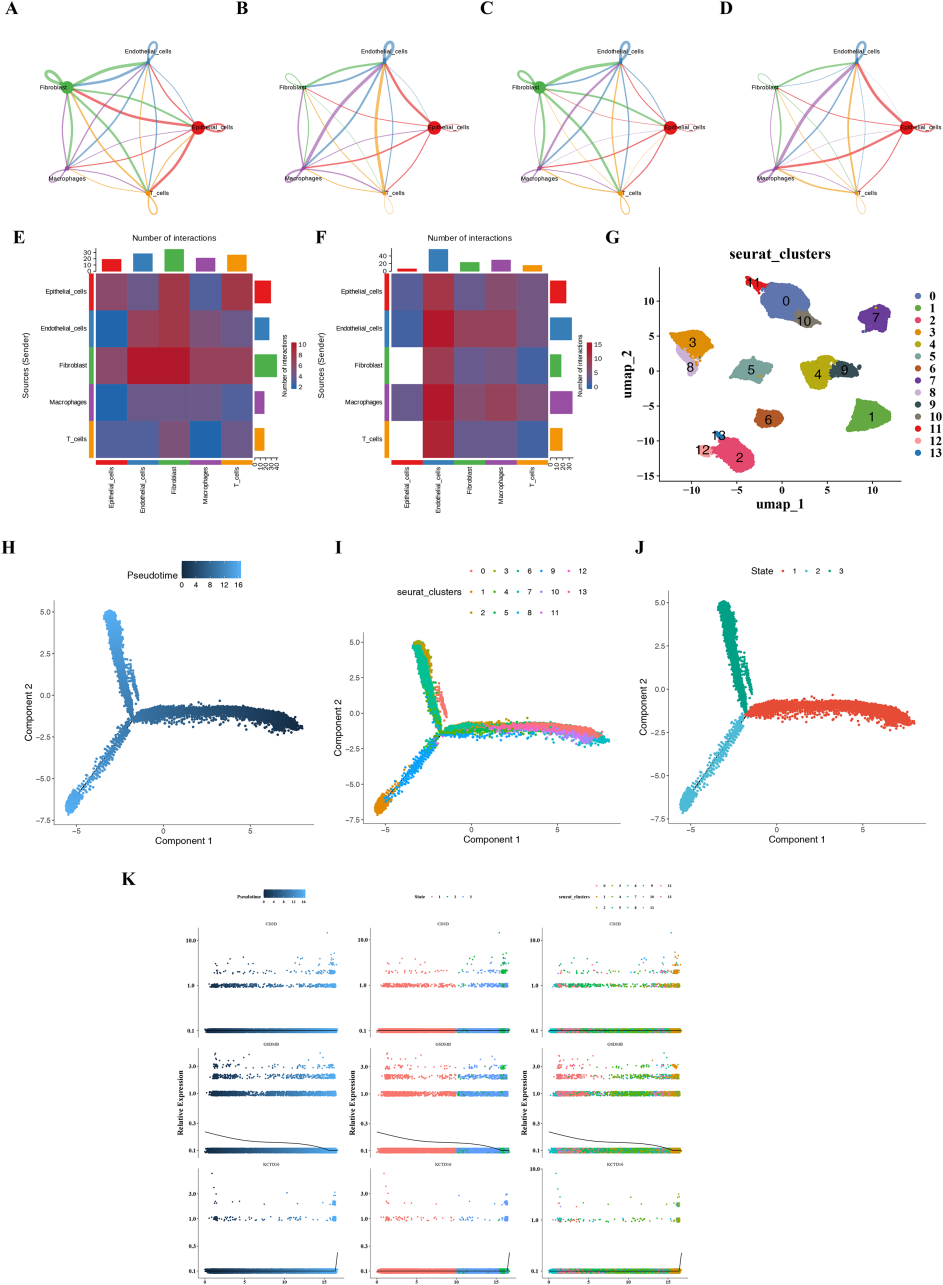
Intercellular communication, developmental trajectory, and experimental validation of prognostic genes in BLCA. **(A, B)** Network diagrams showing the number of interactions between the five main cell types in control group and the BLCA group. The size of the nodes corresponds to the number of cells of each cell type, and the thickness of the edges represents the intensity of the signal interaction between the cell clusters. **(A)** Control group, **(B)** BLCA group. **(C, D)** Network diagram showing the intensity of interactions between the five main cell types in the control group and the BLCA group, **(C)** Control group, **(D)** BLCA group. **(E, F)** Heatmaps illustrating the intensity of cell communication between the five main cell types in the control group and the BLCA group, **(E)** Control group, **(F)** BLCA group. **(G)** UMAP plot of 14 clusters of key epithelial cells after further dimensionality reduction and clustering in the single-cell dataset. **(H–J)** Trajectory plots displaying the pseudo-time analysis results of epithelial subclusters classified by different differentiation times, subgroups and states, **(H)** Differentiation time, **(I)** Differentiation subgroup, **(J)** Differentiation state. **(K)** Expression distributions of prognostic genes in the proposed time trajectory plot. BLCA, bladder cancer; UMAP, Uniform Manifold Approximation and Projection.

In addition, intercellular communication networks were constructed to infer the interactions of different cell types between BLCA and control samples. CellChat analysis revealed the presence of more cellular communication signals between epithelial cells and other populations in BLCA compared to controls (**Figures 8A–D**). In BLCA group, the number of epithelial cells interacting with fibroblasts and T cells was reduced (**Figures 8E, F**). This might have contributed to tumor cells evading immune surveillance and attack, thereby promoting tumor progression. Overall, key cells might play a role in BLCA through intercellular interactions.

### 3.15 Pseudotemporal trajectory of epithelial cell states

In GSE135337, a secondary clustering analysis of epithelial cells was performed, ultimately yielding 14 clusters (**Figure 8G**). Along the inferred pseudotemporal trajectory, cluster 1 was positioned at the endpoint of cell differentiation, whereas cluster 8 marked the starting point. Based on single-cell trajectory analysis, epithelial cells are classified into three differentiation stages. KCTD16 expression remained low until terminal differentiation, while GSDMB showed progressive downregulation along the trajectory (**Figures 8H–K**). Of note, the pseudo-time trajectory delineated herein reflects continuous state transitions of epithelial cells along the pseudo-time axis and does not directly correspond to a linear disease progression from normal to tumor or from non-muscle-invasive to muscle-invasive disease.

To investigate the relationship between epithelial cell differentiation status and tumor infiltration depth, we mapped cells derived from NMIBC and MIBC tissues onto the epithelial differentiation trajectory. Cells from both tumor stages did not preferentially cluster at either terminus of the trajectory but were instead distributed throughout (**Supplementary Figure 7**). Similarly, when Scissor^+^ and Scissor^-^ epithelial cells were projected onto this trajectory, both risk-associated subpopulations were found to contain cells originating from NMIBC and MIBC samples. This observation indicates that the prognostic signature does not simply serve as a surrogate for muscle invasion status, but rather reflects intrinsic molecular characteristics of the tumor. In summary, the pseudo-time analysis presented in this section aims to delineate the dynamic expression patterns of KCTD16 and GSDMB across changing epithelial cell states, rather than to infer a linear progression sequence from normal to tumor or from NMIBC to MIBC.

### 3.16 Validation of prognostic gene expression and cell proliferation

**Figure 9:**
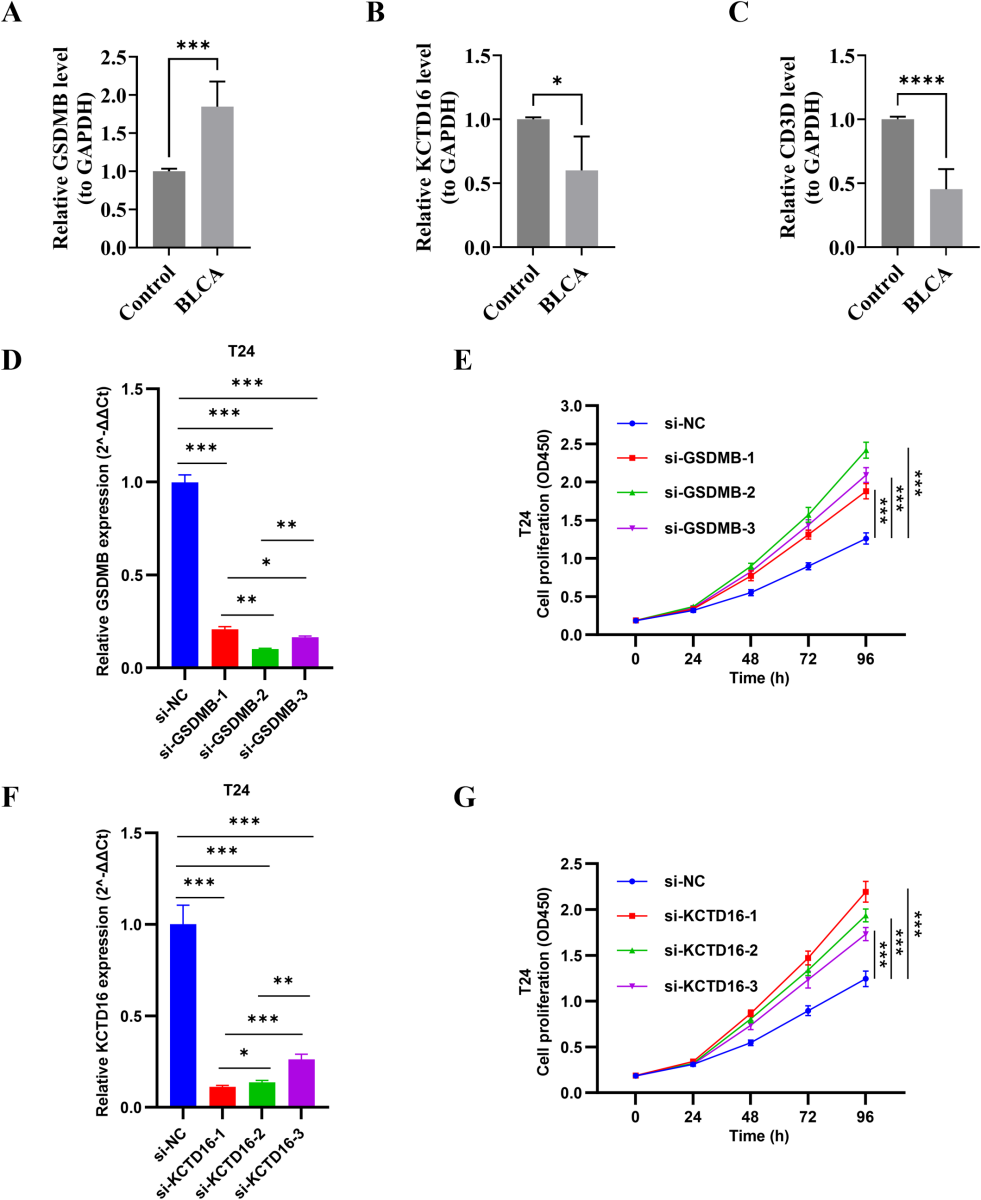
RT-qPCR validation of prognostic genes expression between BLCA and normal tissues, and CCK-8 assay for evaluation of cell proliferation. **(A)** GSDMB. **(B)** KCTD16. **(C)** CD3D. **(D–G)** GSDMB siRNAs and KCTD16 siRNAs were transfected into T24 cells. After incubation for 24, 48, 72, and 96 hours, cell proliferation was conducted using CCK-8 assay. BLCA, bladder cancer; RT-qPCR, quantitative real-time polymerase chain reaction; CCK-8, Cell Counting Kit-8. ^*^p < 0.05; ^**^p < 0.01; ^***^p < 0.001; ^****^p < 0.0001.

RT-qPCR experiments showed that GSDMB was significantly upregulated, while KCTD16 and CD3D were significantly downregulated in BLCA compared to controls (*p* < 0.05) (**Figures 9A–C**). To elucidate the pathological role of GSDMB in BLCA, we first established GSDMB knockdown systems using siRNA in T24 cells. As shown in **Figure 9D**, the knockdown systems were successfully established with high silencing efficiency. CCK−8 assay revealed that GSDMB knockdown significantly enhanced T24 cell proliferation (*p* < 0.05; **Figure 9E**). Similarly, KCTD16 knockdown also significantly enhanced cell proliferation (**Figures 9F, G**). These results suggested that GSDMB, KCTD16, and CD3D might play distinct roles in BLCA pathogenesis and functionally inhibits the proliferation of BLCA cells, thus offering novel perspectives on its molecular mechanisms and potential therapeutic strategies.

## 4 Discussion

BLCA remains one of the most prevalent malignancies of the urinary system, with poor prognosis in advanced stages despite advances in therapeutic strategies (37). Given the urgent need for precise prognostic biomarkers and therapeutic targets, our study focused on succinylation, an emerging post-translational modification that has been implicated in tumor metabolic reprogramming (38) but remains largely unexplored in BLCA. Although individual roles of KCTD16, CD3D, and GSDMB have been previously documented in malignancies, the novelty of the present study lies in the systematic integration of a succinylation-related gene signature with multi-dimensional analyses encompassing TME remodeling, clinical risk stratification, and single-cell transcriptomic landscapes, rather than residing in the mere identification of these genes. By integrating TCGA bulk transcriptomic data with single-cell RNA-seq datasets, we constructed a risk prediction model that demonstrated robust predictive performance across both TCGA and independent GEO validation cohorts (AUC > 0.6). Furthermore, this signature was mechanistically correlated with distinct patterns of TME infiltration, mutational spectra, drug sensitivity profiles, and cell–cell communication networks, underscoring the potential utility of succinylation-associated gene signatures not only as prognostic biomarkers but also as informative tools for guiding therapeutic strategies in BLCA management.

Notably, although our primary training set (TCGA−BLCA) consisted predominantly of MIBC cases, our initial interest in succinylation in BLCA was partly derived from prior evidence linking histone succinylation to NMIBC (8). This distinction does not undermine the validity of our approach, as NMIBC and MIBC lie along a disease continuum, with high−risk NMIBC frequently progressing to MIBC through clonal evolution accompanied by sustained metabolic reprogramming, involving pronounced alterations in lipid and energy metabolism pathways (39, 40). Therefore, extending insights from NMIBC to investigate succinylation−related gene signatures in MIBC provides a rational strategy for discovering biomarkers associated with tumor progression and prognosis, particularly in the absence of large−scale MIBC−specific succinylation datasets.

Our analysis revealed distinct expression and prognostic patterns of the three identified genes. KCTD16, a member of the potassium (K^+^) channel tetramerization domain (KCTD) family containing an N-terminal BTB (Broad complex, Tram-trak and Bric-a-brac)/POZ (poxvirus zinc finger) domain, belongs to the F clade of the KCTD family and functions as an auxiliary subunit of the g-aminobutyric acid (GABA) receptor. KCTD16 exhibits complex and tissue-specific biological functions in cancer research. In papillary thyroid carcinoma, overexpression of KCTD16 is recognized as an independent indicator of poor prognosis (41). In contrast, the expression of KCTD16 is significantly downregulated in lung adenocarcinoma (LUAD), where higher expression levels correlate with improved patient survival, suggesting a potential tumor-suppressive function (42). Consistent with this role, our findings demonstrate that KCTD16 is also significantly downregulated in BLCA, supporting the hypothesis that KCTD16 may act as a tumor suppressor gene in urogenital system malignancies. From a molecular mechanism standpoint, KCTD16 is not involved in the assembly or function of the Cullin3-dependent E3 ubiquitin ligase complex (43). Instead, it may modulate tumor cell proliferation, apoptosis, and migration, as well as the tumor microenvironment, through the regulation of GABA signaling pathways (44). CD3D, an essential component of the T cell receptor (TCR) complex, was also downregulated in BLCA, correlating with reduced T cell infiltration. Consistent with prior studies linking high CD3D expression to better prognosis in BLCA (45) and enhanced responses to immune checkpoint inhibitors in hepatocellular carcinoma (46), our findings suggest that CD3D downregulation in high-risk patients may impair TCR signaling and anti-tumor immunity. Therefore, CD3D has the potential to serve as a valuable biomarker for evaluating the immune status of bladder cancer patients and predicting immunotherapy response.

GSDMB, a gasdermin family member mediating pyroptosis, exhibited upregulated expression in BLCA tissues. However, it showed relatively low expression in the high-risk group defined by our succinylation-related prognostic model, and its knockdown paradoxically promoted the proliferation of T24 cells. This seemingly contradictory phenomenon may be reconciled by considering the functional heterogeneity of GSDMB splice isoforms. Recent studies have established that GSDMB exists as multiple alternatively spliced variants with fundamentally distinct biological activities (47, 48). Isoforms containing exon 6 (GSDMB3/4) can be cleaved by granzyme A to induce pyroptosis and exert anti-tumor effects, whereas isoforms lacking exon 6 (GSDMB1/2) lack the stable belt motif required for membrane insertion and are non-cytotoxic (49). In the context of BLCA, GSDMB is deubiquitinated and stabilized by USP24, after which it directly binds to and activates STAT3. Activated STAT3, in turn, transcriptionally upregulates downstream targets including HK2, LDHA, and IGFBP3, thereby enhancing proliferation, glycolytic metabolism, and immune evasion (50). Therefore, the overall upregulation of GSDMB observed in BLCA tissues likely reflects the predominant expression of non-pyroptotic, pro-tumorigenic isoforms that drive cancer progression via STAT3 pathway activation. Within this mechanistic framework, the relatively lower GSDMB expression detected in the high-risk group may indicate a diminished contribution of this isoform-dependent oncogenic mechanism to the aggressive phenotype of certain advanced tumors. Moreover, the unexpected enhancement of proliferation upon GSDMB knockdown could be attributable to the compensatory activation of alternative proliferative signaling cascades in the absence of the canonical GSDMB–STAT3 axis, thereby paradoxically reinforcing overall proliferative capacity. Collectively, these findings underscore the necessity of distinguishing the functional contributions of specific GSDMB isoforms, rather than relying solely on total transcript abundance, when interpreting its prognostic significance and therapeutic implications in BLCA.

Functional enrichment analysis provides further insights into the biological mechanisms. Our studies identified that KCTD16 and CD3D are co-enriched in GVHD and antigen processing and presentation pathways. Previous studies have indicated that urothelial carcinoma can evade immune surveillance through downregulation of tumor antigen presentation, upregulation of various immune checkpoints, and inactivation of cytotoxic T cells (51). An analysis of BLCA based on TCGA data further demonstrated that subtype cluster 2, characterized by gene signatures associated with antigen processing and presentation, exhibits high CD8^+^ T cell infiltration and high tumor mutational burden, suggesting potential sensitivity to immune checkpoint inhibitor therapy (52). The graft-versus-tumor (GVT) response, one of the most potent forms of cellular immunotherapy, shares immunobiological mechanisms with GVHD, as both involve polyclonal T-cell responses targeting polymorphic antigens presented by recipient hematopoietic tissues (53). Thus, our findings suggest that KCTD16 and CD3D play critical roles in immune recognition and tumor immune evasion, which are consistent with observations in LUAD (54).

GSDMB and CD3D exhibit co-enrichment in the hematopoietic cell lineage pathway. Previous studies have demonstrated notable molecular and functional similarities between BLCA stem cells and normal hematopoietic stem cells (HSCs) (55). Key molecules within the hematopoietic pathway, such as CD44, are also critically involved in promoting proliferation and invasion in bladder cancer (56). Therefore, the co-enrichment of GSDMB and CD3D not only underscores an intrinsic link between BLCA stem cells and hematopoietic-associated molecular mechanisms, but also suggests potential therapeutic targets for overcoming immune evasion and developing targeted strategies directed against this pathway.

Succinylation is a highly conserved lysine acylation modification that utilizes succinyl-CoA, an intermediate metabolite of the tricarboxylic acid (TCA) cycle, as its direct substrate. This modification is primarily catalyzed by succinyltransferases, which covalently attach the succinyl group to lysine residues of target proteins, and can also occur through non−enzymatic reactions (6). Recent studies have revealed that succinylation not only regulates mitochondrial oxidative metabolism but is also widely present on chromatin−associated proteins and histones, thereby establishing a novel epigenetic mark (6, 8, 57). The enrichment of prognostic genes in immune−related pathways (e.g., GVHD, antigen processing and presentation, and hematopoietic cell lineage), may indirectly reflect succinylation−mediated regulation of chromatin accessibility and immune gene transcription. The candidate genes were significantly enriched in potassium ion transmembrane transport, and the activity of potassium channels is known to interactively regulate mitochondrial metabolism and the TCA cycle. Regarding gene expression origin, CD3D is mainly enriched in T lymphocytes, suggesting that its function essentially reflects the level of T cell infiltration and their functional status in the tumor microenvironment, rather than representing intrinsic characteristics of tumor epithelial cells. In contrast, KCTD16 and GSDMB exhibit dynamic expression changes in epithelial cells, indicating that succinylation−related regulation may directly contribute to the malignant transformation of epithelial cells. Thus, the above functional enrichment analyses propose a landscape in which succinylation coordinates metabolic reprogramming, epigenetic remodeling, and immune modulation to influence bladder cancer progression. And our model integrates tumor-intrinsic traits and immune status, forming the biological basis for robust prognosis. Moreover, immune microenvironment analysis showed that high-risk patients had lower immune scores and higher TIDE scores, suggesting stronger immune evasion and reduced responsiveness to PD-1/PD-L1 inhibitors, consistent with observations in endometrial cancer (58). The higher TP53 mutation rate in the high-risk group further reinforced the connection between risk stratification and immune escape mechanisms (59).

Drug sensitivity analysis revealed stratified therapeutic implications. High-risk patients were more sensitive to the PLK1 inhibitor BI-2536_1086, consistent with the essential role of PLK1 in cell cycle regulation and chemotherapy sensitization (60, 61). In contrast, low-risk patients exhibited enhanced sensitivity to conventional chemotherapeutics such as cisplatin and paclitaxel, potentially due to higher immune infiltration synergizing with chemotherapy, a mechanism previously observed in gastric cancer (62). These findings suggest that high-risk BLCA patients may benefit from targeted strategies, whereas low-risk patients could respond better to chemo-immunotherapy combinations, providing a new perspective for a deeper understanding of the molecular basis of immune therapy resistance in BLCA.

Single-cell analysis provided additional resolution by uncovering cell–type–specific expression patterns. The bladder epithelium plays crucial roles in maintaining immune surveillance, facilitating injury repair, and participating in the initiation and progression of tumors. Under pathological conditions such as infection or damage, quiescent epithelial stem cells can re-enter the cell cycle to promote tissue regeneration and repair (63). Normal urothelial cells contribute to immune surveillance through mechanisms such as “antigen disparity,” which can trigger host immune responses by recruiting cytotoxic immune cells to eliminate abnormal and early-stage tumor cells (63). Furthermore, distinct subpopulations of epithelial cells demonstrate divergent biological behaviors (64). Basal cells, possessing intrinsic epithelial-mesenchymal transition (EMT) traits, drive tumor invasion into deeper tissues at the T1 stage, and the luminal subtypes facilitates continuous tumor growth and expansion of micro-metastatic foci by sustaining high proliferative activity. Meanwhile, the muscle-invasive tumor acquires enhanced self-renewal capacity through SOX9/SOX2-mediated stem cell-like properties, markedly augmenting tumor malignancy and treatment resistance. KCTD16 and CD3D were most differentially expressed in epithelial cells, implicating these cells as key targets of succinylation. The Scissor algorithm revealed that both Scissor^+^ and Scissor^-^ cells were mainly distributed within epithelial cell populations rather than in immune or stromal cells, suggesting that transcriptomic heterogeneity in epithelial cells may represent the primary cytological basis influencing patient prognosis. Furthermore, remarkable functional heterogeneity exists within the tumor epithelial cell population—some epithelial cell subpopulations exhibit transcriptional profiles closely related to poor prognosis (Scissor^+^), while others are associated with favorable outcomes (Scissor^-^). Consistent with above findings that epithelial cells are critical players, these results further identify the existence of prognostic risk-related functional subpopulations within epithelial cells. In-depth analysis of the Scissor^+^ subpopulation in epithelial cells is expected to provide a novel perspective for elucidating the cellular mechanisms driving BLCA malignancy and to offer a theoretical basis for future targeted therapeutic strategies directed against specific epithelial subpopulations.

Aberrant ligand–receptor interactions between epithelial cells, fibroblasts, and T cells indicated disrupted intercellular communication, similar to pancreatic cancer, where tumor–stroma signaling promotes immune escape (65). Pseudotime analysis revealed dynamic regulation, with KCTD16 expression increasing at late epithelial differentiation stages, while GSDMB decreased progressively, suggesting their roles in differentiation and epithelial–mesenchymal transition (66). The low detection of KCTD16 in single−cell UMAP visualization arises from technical dropout and inefficient capture of low-abundance transcripts in scRNA-seq, whereas its prognostic significance stems from its differentiation-dependent upregulation in rare but functionally critical malignant epithelial subpopulations and the superior sensitivity of bulk RNA-seq to such minor cell signals. RT−qPCR was used to validate the expression of three genes in cancer and adjacent normal tissues. CCK−8 assays at the cellular level confirmed that GSDMB and KCTD16 function as tumor suppressor genes (excluding CD3D, a T−cell marker gene), which is consistent with their HR values of less than 1 in the Cox regression analysis.

This study presents the first succinylation-related prognostic model for BLCA, integrating multi-omics and single-cell transcriptomic analysis. The identified genes—KCTD16, CD3D, and GSDMB—were associated with prognosis, immune microenvironment modulation, drug sensitivity, and intercellular communication, underscoring their translational potential as biomarkers and therapeutic targets. Nevertheless, some limitations remain. First, this study was based on transcriptomic mRNA data without directly detecting target protein succinylation at the proteomic or succinylomic levels, thus failing to reflect the actual succinylation status or the regulatory link between transcription and protein modification. Second, *in vitro* and *in vivo* experiments are needed to validate the roles of KCTD16, CD3D, and GSDMB in BLCA, which would further elucidate the molecular mechanisms and strengthen our conclusions. Third, differences exist in the analysis strategies between TCGA-BLCA and Soochow-BLCA: the former was based on stratification of 20 SRGs, whereas the latter—limited by the sample size of the local cohort— only compared tumors with normal tissues without adopting a unified strategy. This to some extent affects the comparability of the results. Fourth, the root cells of the pseudo-time trajectory are automatically selected by the algorithm and lack biological validation. Finally, due to the inherent biases in publicly available datasets, prospective validation using large-scale, multi-center clinical cohorts is essential to establish clinical utility prior to any potential clinical application. Future research will focus on multi-omics integration (transcriptomic, proteomic, and succinylome), mechanistic characterization of core genes via *in vitro* and *in vivo* models, enhanced pseudo-time trajectory reliability through RNA velocity combined with experimental calibration, expanded local Soochow-BLCA cohort validation, and refined epithelial subpopulation classification. These efforts will ultimately provide a more comprehensive understanding of succinylation in BLCA and pave the way for precision therapeutic strategies.

## 5 Conclusion

In conclusion, this study established a comprehensive bioinformatics framework to identify succinylation-related prognostic genes in BLCA and developed a robust prognostic signature based on three core genes—KCTD16, CD3D, and GSDMB—selected via machine learning algorithms. The risk model demonstrated consistent predictive accuracy across independent cohorts and was significantly associated with immune microenvironment alterations, mutational profiles, and differential drug sensitivity. Functional investigations revealed the involvement of these genes in immune and hematopoietic pathways, while single-cell RNA sequencing highlighted epithelial cells as key players exhibiting altered intercellular communication and pseudotemporal expression dynamics. Experimental validation further confirmed the dysregulation of these genes in BLCA tissues. Our findings provide novel insights into the roles of succinylation in BLCA pathogenesis and progression, offering a potential biomarker system for prognosis prediction and tailored therapeutic strategies. Further mechanistic studies and clinical validations are warranted to translate these findings into clinical practice.

## Data Availability

The data presented in the study are deposited in the TCGA and GEO repository, with accession number of GSE13507 and GSE135337. Given privacy, ethical and legal considerations, the transcriptome sequencing data along with relevant clinical information from the Soochow-BLCA cohort (15 pairs of BLCA and adjacent normal tissue samples obtained from the Fourth Affiliated Hospital of Soochow University) will be made available by the corresponding author upon reasonable request.

## Supplementary material

Supplementary Figure 1 Univariable Cox regression analysis of the 20 SRGs. **(A)** Proportional hazards test. **(B)** Univariable Cox regression forest plot. The horizontal line represents the 95% confidence interval. HR > 1 indicates a risk factor, and HR < 1 indicates a protective factor. SRGs, succinylation-related genes.

Supplementary Figure 2 Violin plot displaying associations between risk score and various clinical features in TCGA-BLCA training set. **(A)** Clinical stage of BLCA. **(B)** Tumor stage. **(C)** Node stage. **(D)** Metastasis stage. **(E)** Age. **(F)** Gender. **(G)** Heatmap showing the expression differences of prognostic genes in NMIBC and MIBC tissues. Each column represents a sample, and each row represents a gene. The color range from blue to red indicates the expression levels from low to high. BLCA, bladder cancer; NMIBC, non-muscle-invasive bladder cancer; MIBC, muscle-invasive bladder cancer. ^*^p < 0.05; ^**^p < 0.01; ^***^p < 0.001; ^****^p < 0.0001; ns, no significant.

Supplementary Figure 3 Correlation analysis of key genes with 20 SRGs. SRGs, succinylation-related genes.

Supplementary Figure 4 Quality control and feature selection of scRNA-seq data in the validation cohort of GSE135337. **(A)** Violin plots showing the distribution of nFeature_RNA, nCount_RNA, and mitochondrial gene percentage after quality filtering. **(B)** Scatter plot demonstrating strong correlation between nCount_RNA and nFeature_RNA across cells. **(C)** Scree plot visualizing top 2000 HVGs with top 10 most variable genes labeled. **(D)** Elbow plot visualizing the standard deviations of PCs. **(E)** JackStraw plot showing statistical significance of the top 20 PCs. scRNA-seq, single-cell RNA sequencing; BLCA, bladder cancer; nFeature_RNA, number of genes detected per cell; nCount_RNA, total RNA counts per cell; HVGs, highly variable genes; PCs, principal components.

Supplementary Figure 5 Comparison of sample origin and disease stage distribution in the single-cell UMAP map of BLCA. **(A)** Classification of cell clusters in BLCA and normal tissue. **(B)** UMAP map of cell distribution based on different sample origins and tumor stages. BLCA, bladder cancer; UMAP, Uniform Manifold Approximation and Projection.

Supplementary Figure 6 Scissor algorithm identifies cell subpopulations associated with prognosis risk. **(A)** Statistical count of Scissor^+^ cells (associated with high risk) and Scissor^-^ cells (associated with low risk). **(B)** UMAP dimensionality reduction plot showing the spatial distribution of the three types of Scissor cells in the single-cell dataset GSE135337. **(C)** Absolute number and relative proportion of Scissor^+^ cells in each major cell type. **(D)** Cell-type-specific distribution of Scissor^+^ and Scissor^-^ phenotypes in NMIBC and MIBC. UMAP, Uniform Manifold Approximation and Projection; NMIBC, non-muscle-invasive bladder cancer; MIBC, muscle-invasive bladder cancer.

Supplementary Figure 7 Pseudotime distribution scatter plot of MIBC and NMIBC samples. The scatter plot displays Component 1 on the x-axis and Component 2 on the y-axis, with colors representing the two groups along the differentiation trajectory. NMIBC, non-muscle-invasive bladder cancer; MIBC, muscle-invasive bladder cancer.

## Glossary

BLCA: bladder cancer
CI: confidence interval
NMIBC: non-muscle-invasive bladder cancer
MIBC: muscle-invasive bladder cancer
TURBT: transurethral resection of bladder tumor
OS: overall survival
scRNA-seq: single-cell RNA sequencing
SRGs: succinylation-related genes
DEGs: differentially expressed genes
FC: fold change
ssGSEA: single-sample Gene Set Enrichment Analysis
K-M: Kaplan-Meier
GO: Gene Ontology
KEGG: Kyoto Encyclopedia of Genes and Genomes
PPI: protein-protein interaction
HR: Hazard Ratio
PH: proportional hazards
LASSO: least absolute shrinkage and selection operator
HRG: high-risk group
LRG: low-risk group
ROC: receiver operating characteristic
BP: biological process
CC: cellular component
MF: molecular function
NES: normalized enrichment score
GVHD: graft versus host disease
TME: tumor microenvironment
TIDE: Tumor Immune Dysfunction and Exclusion
TMB: tumor mutation burden
GDSC: Genomics of Drug Sensitivity in Cancer
IC_50_: half-maximal inhibitory concentration
HVGs: highly variable genes
PCs: principal components
UMAP: Uniform Manifold Approximation and Projection
RT-qPCR: quantitative real-time polymerase chain reaction
KCTD: Potassium (K^+^) Channel Tetramerization Domain
BTB: Broad complex, Tram-trak and Bric-a-brac
POZ: poxvirus zinc finger
GABA: g-aminobutyric acid
LUAD: lung adenocarcinoma
TCR: T cell receptor
GVT: graft-versus-tumor
HSCs: hematopoietic stem cells
EMT: epithelial-mesenchymal transition
