# Supplementary figures and images for: Identification of succinylation-related genes in bladder cancer: integration of single-cell and transcriptomic data
Source: Front Immunol. 2026 May 15;17:1797389. doi: 10.3389/fimmu.2026.1797389 (PMC13218921; doi:10.3389/fimmu.2026.1797389)

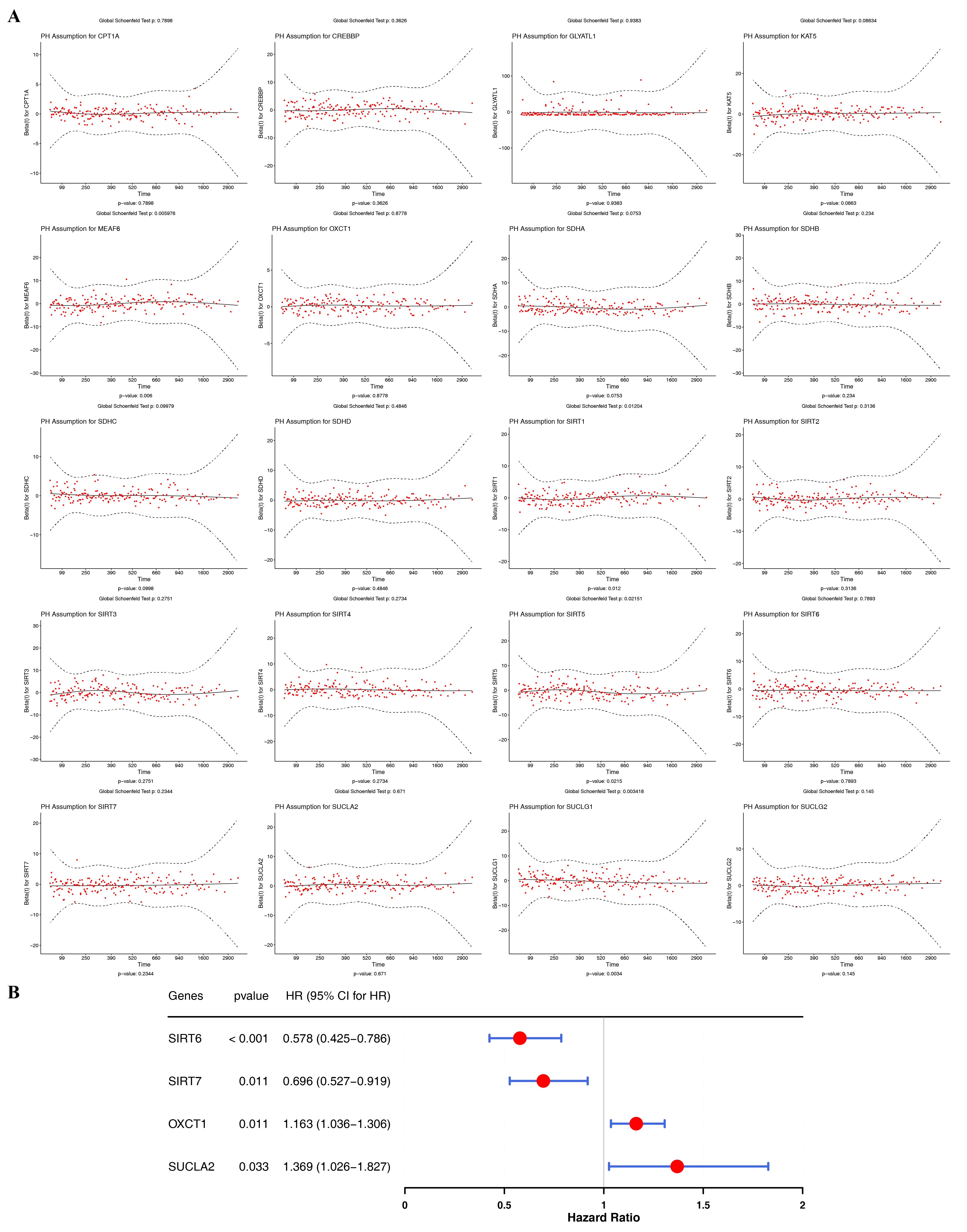

Supplement: Supplementary Figure 1 — Univariable Cox regression analysis of the 20 SRGs. (A) Proportional hazards test. (B) Univariable Cox regression forest plot. The horizontal line represents the 95% confidence interval. HR > 1 indicates a risk factor, and HR < 1 indicates a protective factor. SRGs, succinylation-related genes. [file Image1.tif]

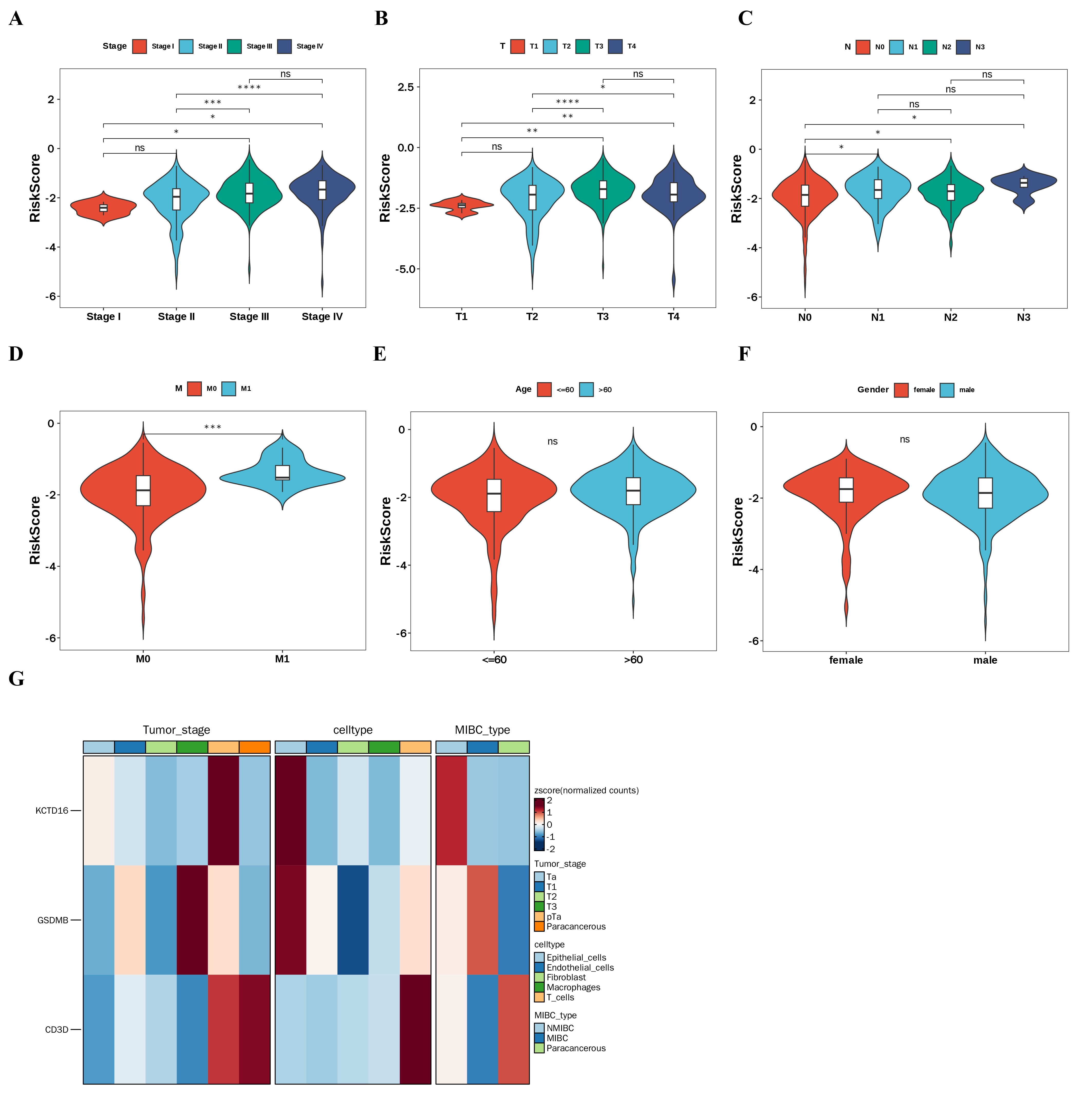

Supplement: Supplementary Figure 2 — Violin plot displaying associations between risk score and various clinical features in TCGA-BLCA training set. (A) Clinical stage of BLCA. (B) Tumor stage. (C) Node stage. (D) Metastasis stage. (E) Age. (F) Gender. (G) Heatmap showing the expression differences of prognostic genes in NMIBC and MIBC tissues. Each column represents a sample, and each row represents a gene. The color range from blue to red indicates the expression levels from low to high. BLCA, bladder cancer; NMIBC, non-muscle-invasive bladder cancer; MIBC, muscle-invasive bladder cancer. *p < 0.05; **p < 0.01; ***p < 0.001; ****p < 0.0001; ns, no significant. [file Image2.tif]

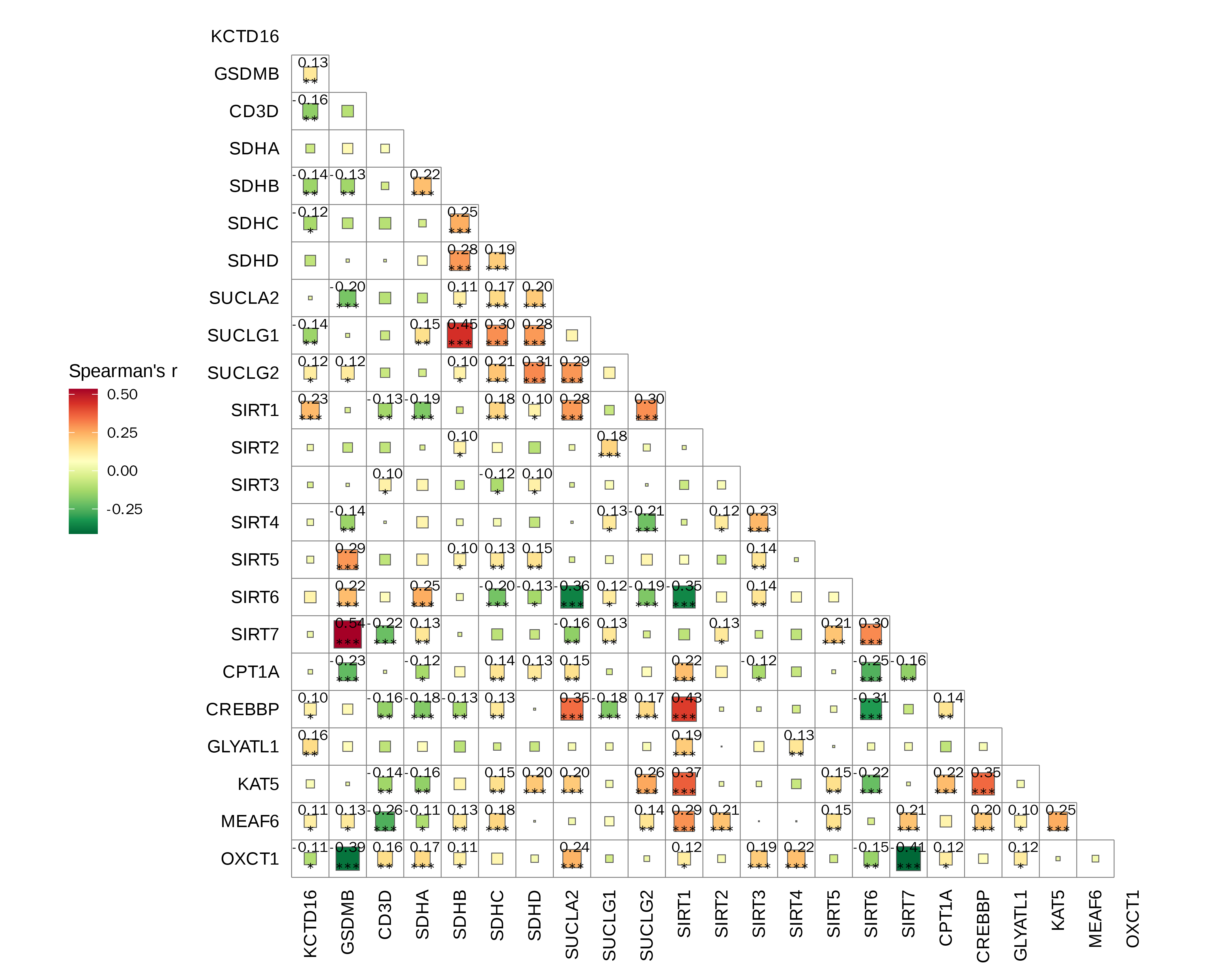

Supplement: Supplementary Figure 3 — Correlation analysis of key genes with 20 SRGs. SRGs, succinylation-related genes. [file Image3.tif]

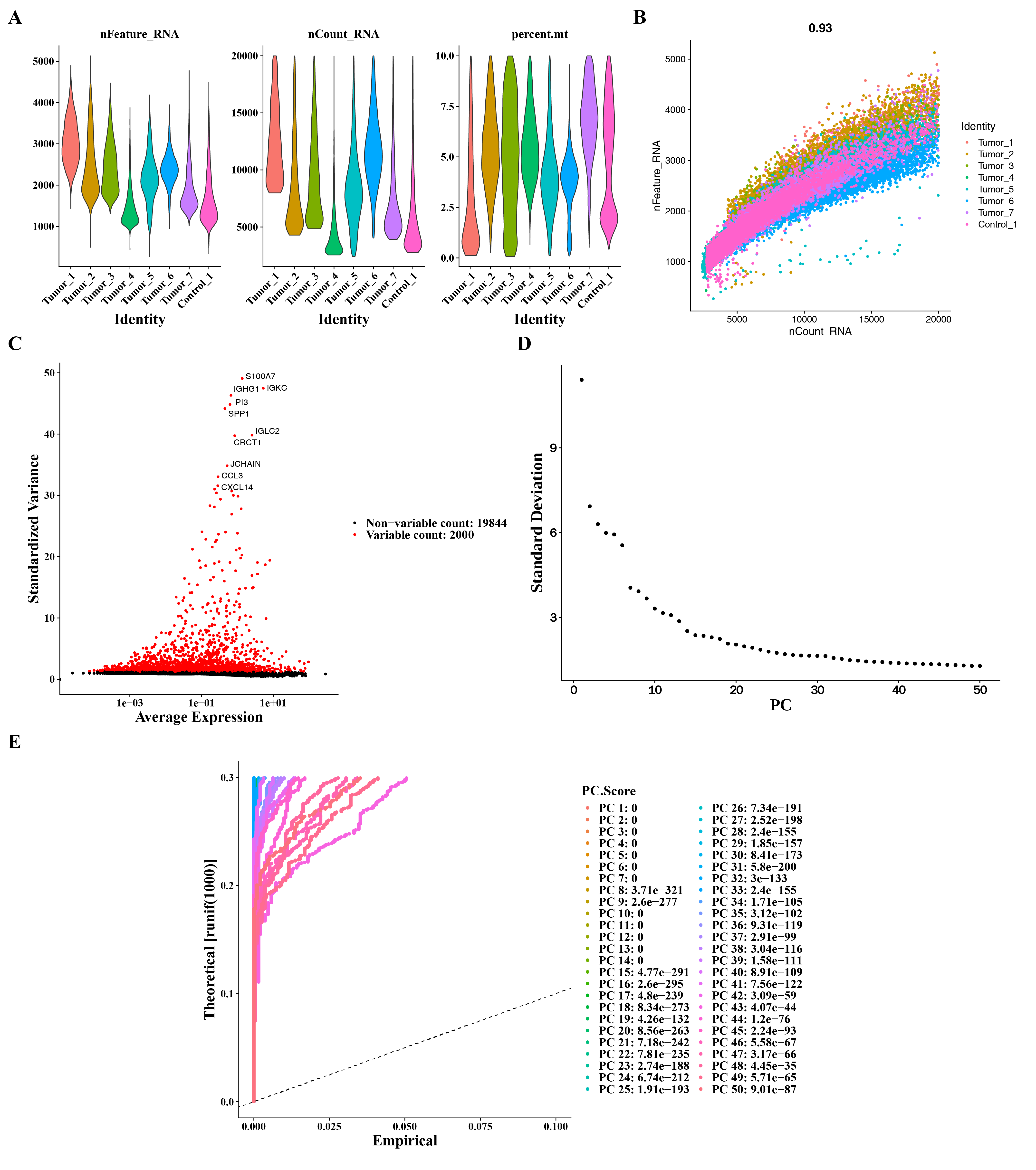

Supplement: Supplementary Figure 4 — Quality control and feature selection of scRNA-seq data in the validation cohort of GSE135337. (A) Violin plots showing the distribution of nFeature_RNA, nCount_RNA, and mitochondrial gene percentage after quality filtering. (B) Scatter plot demonstrating strong correlation between nCount_RNA and nFeature_RNA across cells. (C) Scree plot visualizing top 2000 HVGs with top 10 most variable genes labeled. (D) Elbow plot visualizing the standard deviations of PCs. (E) JackStraw plot showing statistical significance of the top 20 PCs. scRNA-seq, single-cell RNA sequencing; BLCA, bladder cancer; nFeature_RNA, number of genes detected per cell; nCount_RNA, total RNA counts per cell; HVGs, highly variable genes; PCs, principal components. [file Image4.tif]

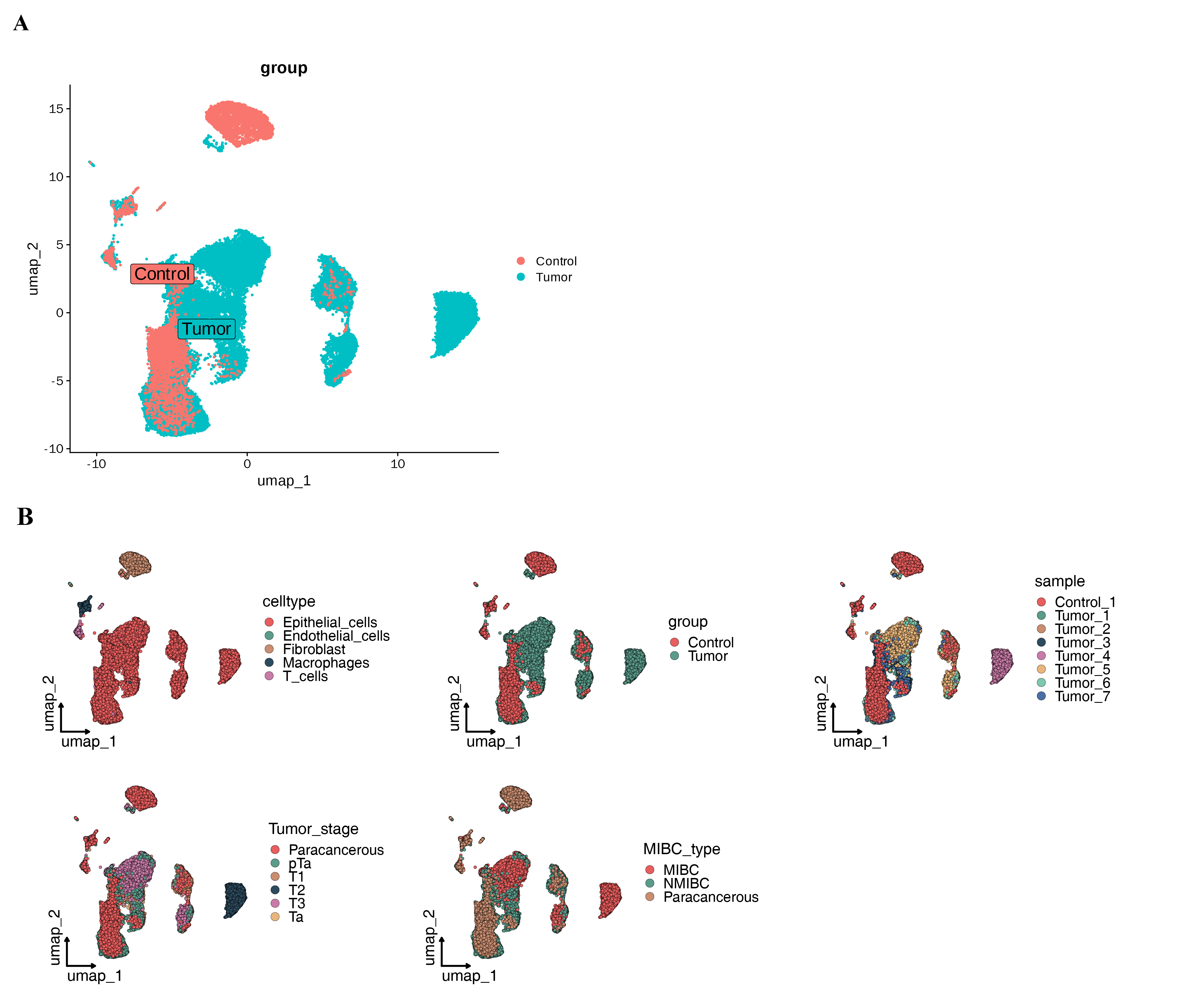

Supplement: Supplementary Figure 5 — Comparison of sample origin and disease stage distribution in the single-cell UMAP map of BLCA. (A) Classification of cell clusters in BLCA and normal tissue. (B) UMAP map of cell distribution based on different sample origins and tumor stages. BLCA, bladder cancer; UMAP, Uniform Manifold Approximation and Projection. [file Image5.tif]

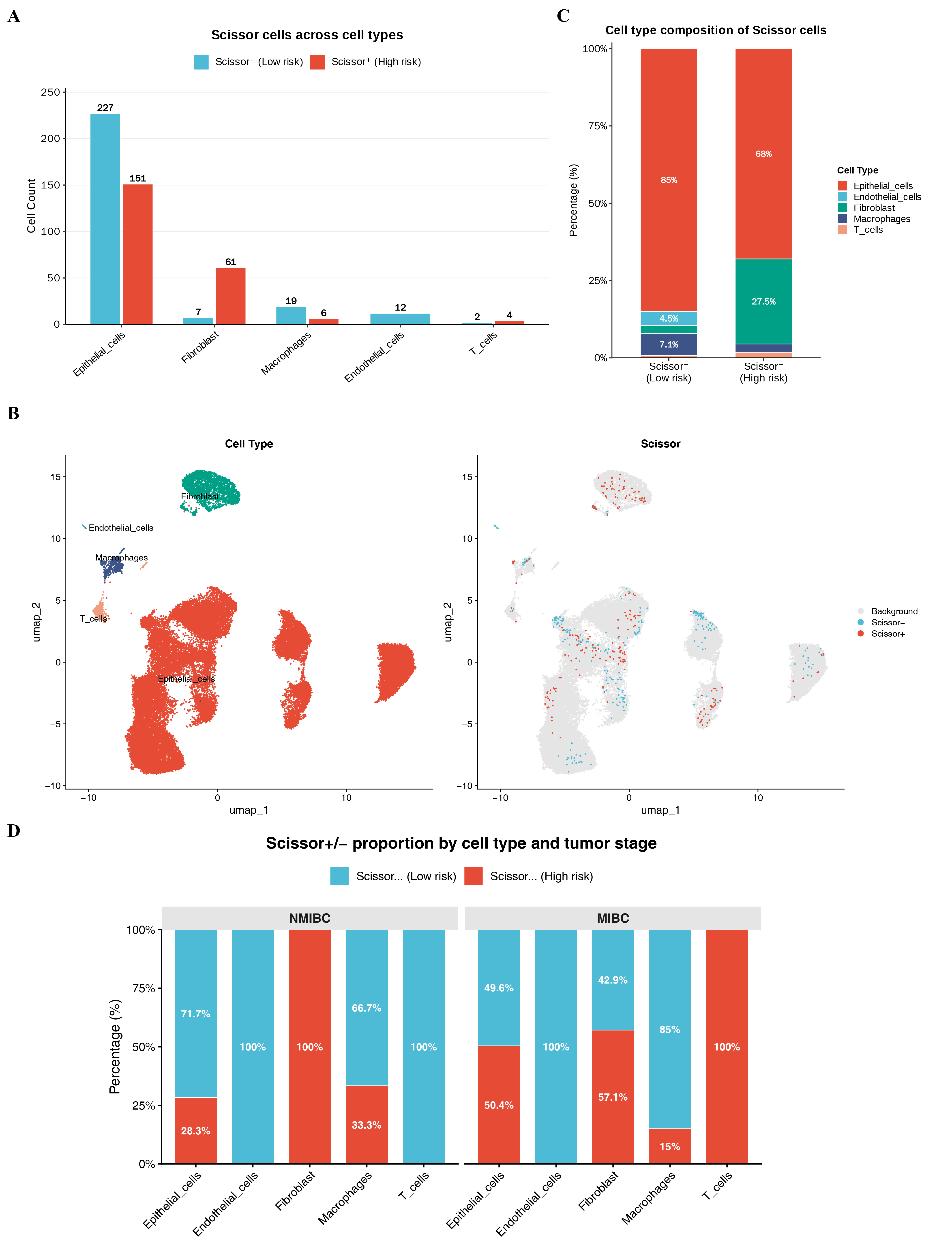

Supplement: Supplementary Figure 6 — Scissor algorithm identifies cell subpopulations associated with prognosis risk. (A) Statistical count of Scissor+ cells (associated with high risk) and Scissor- cells (associated with low risk). (B) UMAP dimensionality reduction plot showing the spatial distribution of the three types of Scissor cells in the single-cell dataset GSE135337. (C) Absolute number and relative proportion of Scissor+ cells in each major cell type. (D) Cell-type-specific distribution of Scissor+ and Scissor- phenotypes in NMIBC and MIBC. UMAP, Uniform Manifold Approximation and Projection; NMIBC, non-muscle-invasive bladder cancer; MIBC, muscle-invasive bladder cancer. [file Image6.tif]

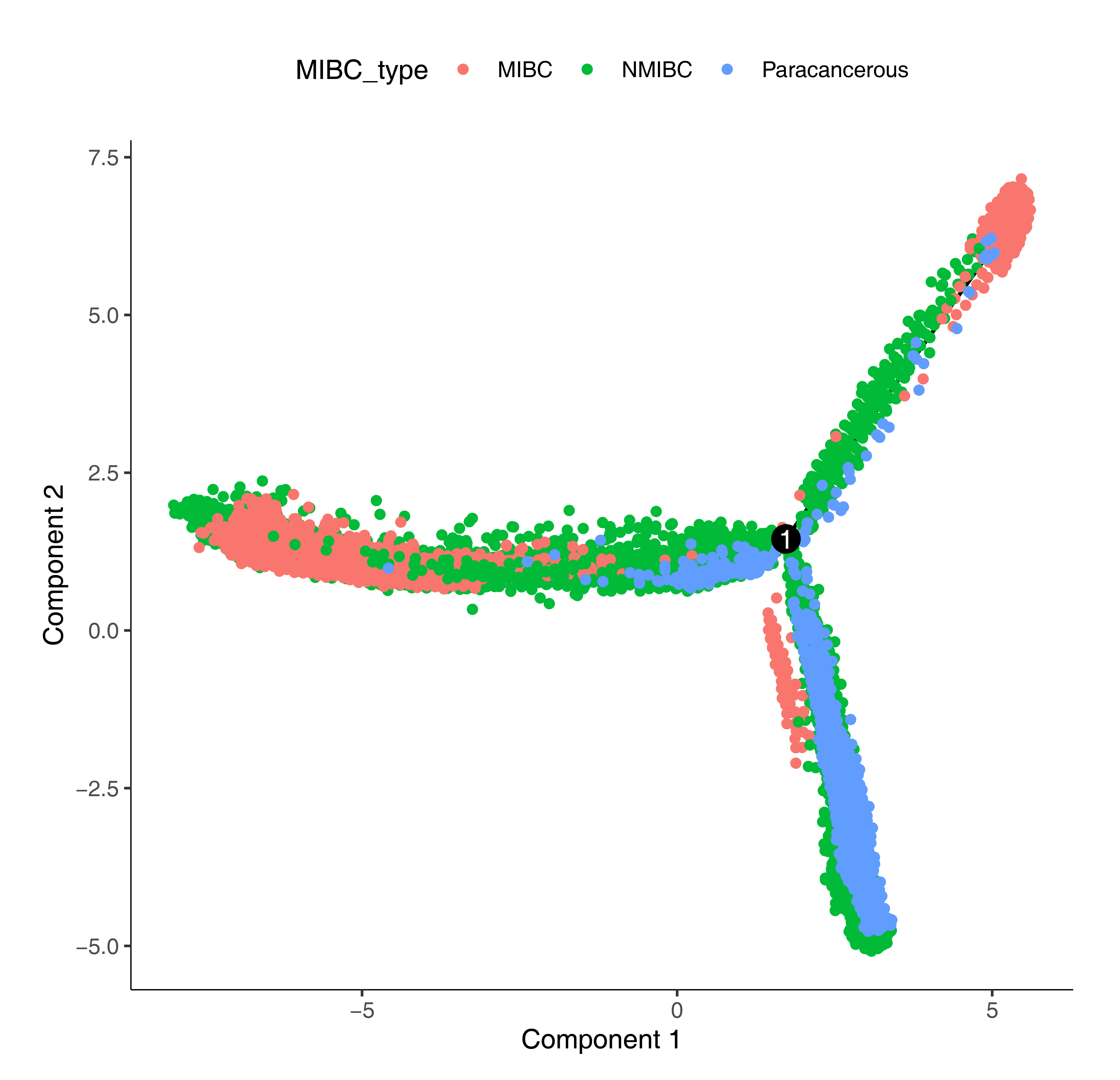

Supplement: Supplementary Figure 7 — Pseudotime distribution scatter plot of MIBC and NMIBC samples. The scatter plot displays Component 1 on the x-axis and Component 2 on the y-axis, with colors representing the two groups along the differentiation trajectory. NMIBC, non-muscle-invasive bladder cancer; MIBC, muscle-invasive bladder cancer. [file Image7.tif]
